# Vaccinology in sub-Saharan Africa

**DOI:** 10.1136/bmjgh-2018-001363

**Published:** 2019-09-20

**Authors:** Jennifer Moïsi, Shabir Ahmed Madhi, Helen Rees

**Affiliations:** 1 Independent Consultant, Paris, France; 2 Department of Science and Technology/National Research Foundation: South African Research Chair Initiative: Vaccine Preventable Diseases—Medical Research Council: Respiratory and Meningeal Pathogens Research Unit, Johannesburg, Gauteng, South Africa; 3 African Leadership Initiative for Vaccinology Expertise, University of the Witwatersrand, Johannesburg, Gauteng, South Africa; 4 Wits Reproductive Health and HIV Institute, Johannesburg, Gauteng, South Africa

**Keywords:** vaccinology, immunization, sub-saharan africa, capacity building

## Abstract

We undertook a landscape analysis of vaccinology research and training in sub-Saharan Africa in order to identify key gaps and opportunities for capacity development in the field. We conducted interviews with regional and global immunisation experts, reviewed university and research centre websites, searched the scientific literature and analysed donor databases as part of our mapping exercise. We found that (1) few vaccinology training programmes are available in the region; (2) vaccinology research sites are numerous but unevenly distributed across countries and subregions and of widely varying capacity; (3) donor funding favours HIV, tuberculosis and malaria vaccine development over other high-burden diseases; (4) lack of vaccine design, manufacturing and regulatory capacity slows the progress of new vaccines through the research and development pipeline and (5) vaccine implementation research garners limited support. Regional efforts to strengthen African vaccinology expertise should develop advanced vaccinology training programmes, support clinical trial and implementation research sites in geographic areas with limited capacity and conduct multidisciplinary research to help design, license and roll out new vaccines.

**Summary box**

- A large number of sites conduct vaccinology research in sub-Saharan Africa, but they vary widely in size and capacity and are heterogeneously distributed across the region.
- Training opportunities for African vaccinologists are limited.
- Donor funding remains overwhelmingly focused on HIV, tuberculosis and malaria vaccine development, dwarfing investment in high-burden diseases such as respiratory infections and enteric illnesses.
- The pipeline of new products targeting priority diseases for Africa is extensive, but rapid development is hampered by the lack of vaccine design, manufacturing and regulatory capacity in the region.
- There is little investment in an implementation science agenda that would increase immunisation programme efficiency and effectiveness in different settings.

## Introduction

The UN Sustainable Development Goals (SDGs) set out ambitious targets for the global community over the period 2015–2030. SDG3 focuses on good health and well-being, aiming to ‘*support the research and development of vaccines and medicines for the communicable and non-communicable diseases that primarily affect developing countries (and) provide access to affordable essential medicines and vaccines’.* 1 This goal was reaffirmed in the 2016 Addis declaration on Universal Access to Immunization as a Cornerstone for Health and Development in Africa, which asserted high-level country support for strengthening immunisation programmes to reach the most vulnerable populations.^2^ In order to meet these objectives, the effectiveness and programmatic suitability of existing vaccines need to be investigated and improved.

Ongoing or completed evaluations of the immunogenicity of reduced dosing schedules for pneumococcal conjugate vaccines,^3 4^ of the operational feasibility, safety and impact of delivering RTS, S-AS01 vaccine within the Expanded Programme for Immunisation (EPI)^5^ and of the safety and immunogenicity of fractional doses of yellow fever vaccine for epidemic response^6 7^ highlight the importance of implementation research to immunisation programmes. In parallel to this strong operational focus, international support for new vaccine development is rising, driven by the imperative to prevent long-standing public health problems, such as tuberculosis and influenza. The 2014–2015 West African Ebola epidemic and rising antimicrobial resistance demonstrate the need for rapid vaccine development pathways to target emerging pathogens with epidemic potential^8^ and combat the spread of multidrug-resistant pathogens.^9^ Donors, development partners and countries are therefore working together to address the two separate objectives of strengthening programmes to improve population access to existing vaccines and developing new vaccines for global populations.

In this context, we undertook a landscape analysis to describe the global and African vaccinology environment and identify key gaps and opportunities for building research and training capacity in the region, with the ultimate aim of accelerating vaccine discovery and clinical evaluation and providing evidence to improve immunisation policy and programmes. (The African Leadership Initiative for Vaccinology Expertise (ALIVE) was established in 2016 as a South African Department of Science and Technology/National Research Foundation Flagship Initiative at the University of Witwatersrand, Johannesburg. This landscape analysis was conducted in preparation for the development of the ALIVE strategy and workplan.)

## Vaccinology training

We collected detailed information on 608 universities in 48 countries (online supplementary methods and online supplementary table 1). We found that institutional density (number of universities per 10 million population) varies widely across countries and African subregions (online supplementary figures 1-3). When related to population size, the median number of universities is 2.1 (IQR 1.0–4.6) per 10 million people for biological sciences, 2.4 (IQR 1.4–5.1) per 10 million for medicine and 1.7 (IQR 0.2–3.7) per 10 million for public health, with 10-fold differences in density across the region. We also showed that basic science training is more widely available than social science: while most countries have at least one institution that conducts research or offers training in microbiology (n=42/48), virology, immunology and epidemiology are not as commonly found (n=34, 37 and 38/48, respectively) and health economics and health policy are frequently non-existent (n=24 and 22/48 countries have programmes, respectively).

10.1136/bmjgh-2018-001363.supp12 Supplementary data

10.1136/bmjgh-2018-001363.supp1 Supplementary data

This analysis of university data provides a high-level description of training available across the continent and helps to understand the disciplines that vaccinology trainees could have exposure to during their undergraduate or graduate studies. Nevertheless, it has some important limitations. First, there are likely inaccuracies in the information collected from university websites, which may be incomplete, out of date or imprecise. Second, we were unable to access information on the number of students trained annually and on the number of students who have emigrated, limiting our ability to interpret the data in terms of population impact: as an example, Somalia and Sudan have a high density of medical schools but a very low density of physicians,^10^ demonstrating the lack of correlation between the number of institutions and the number of physicians trained and remaining in country to practise medicine. Finally, and perhaps most importantly, we have no information on the quality of training offered: evidence from our expert interviews suggests that laboratory technician degree programmes in some West African countries do not include any bench instruction, so graduates are unable to perform basic tasks in their field unless they receive further professional training.

In addition to general information on scientific and medical education in sub-Saharan Africa, we searched specifically for ‘vaccinology’ training programmes. Only 10 ‘vaccinology’ courses are currently offered in the region (table 1). These courses are of varying durations and levels and include five short courses focused on basic vaccinology concepts and programme implementation issues, targeting health professionals (clinicians, pharmacists) or immunisation managers; two short courses with strong scientific and policy components, which are more advanced and aim to engage scientists, covering vaccine design, development and policy and three 9–18 month long Masters-level programmes, varying in focus from biomedical to managerial. African scientists may also participate in various short courses or degree programmes offered in Europe or North America such as ADVAC (Fondation Mérieux/University of Geneva), Masters in Vaccinology and Clinical Development (University of Siena) or Summer School on Vaccinology (University of Antwerp). However, the advantages of participating in an Africa-based course or training programme go beyond the reduced travel distance and lower cost. The focus on African vaccinology topics, the ability to interact with colleagues from the region with a variety of experiences and backgrounds and the opportunity to develop a network that will form the basis for future partnerships is a distinct advantage that only Africa-based and Africa-tailored training can provide.

**Table 1:** Vaccinology courses in sub-Saharan Africa

Name of course	Organiser	Duration	Language	Frequency	Target audience	Comments
Regional vaccinology course	WHO Regional Office for Africa (AFRO)	2 weeks	French, English, Portuguese	Annual, alternate languages	Academics, clinicians, National Technical Advisory Group members	Partnership with various academic institutions
Mid-Level Management (MLM) course on the Expanded Programme on Immunisation (EPI)	WHO AFRO	2 weeks	French, English, Portuguese	Annual, alternate languages	Immunisation programme managers	Partnership with various academic institutions; adapted for cascade training
Vaccinology in Africa	Jenner Institute, University of Oxford, UK	5 days	English	Annual	Scientists interested in vaccine research and development	Partnership with African host institution
Masters International en Vaccinologie Appliquée	Agence de Médecine Préventive/Université Paris Dauphine, France	4 weeks in person, 9 months distance training+thesis	French	Annual	Immunisation programme managers with medical degree or masters of public health.	Partnership with universities in Benin, Burkina Faso, Côte d’Ivoire, Togo Epivac.net network of alumni
Diplôme Inter- Universitaire International de Vaccinologie	Université polytechnique de Bobo-Dioulasso, Burkina Faso	2 weeks in person+thesis	French	Annual	Physicians, pharmacists, health professionals with at least bachelors degree	
Vaccinology course	Kenya AIDS Vaccine Initiative Institute of Clinical Research at University of Nairobi, Kenya	5 days	English	At least annual	Clinicians, lab scientists, programme managers	
Vaccinology course for Health Professionals	East Africa Center for Vaccines and Immunization	5 days	English	At least annual	Health professionals, specifically vaccine handlers	Partnership with universities, paediatric associations and EPI programme in Kenya, Tanzania Uganda
Masters in Infectious Diseases and Vaccinology	Jomo Kenyatta University of Agriculture and Technology, Kenya	18 months	English	Annual intake	Individuals with bachelors degree in biological science, medicine, pharmacy	
African Advanced Course in Vaccinology	Respiratory and Meningeal Pathogens Research Unit and National Institute for Communicable Diseases, South Africa	10 days	English	Biennially in Africa	Immunisation programme managers, researchers and public health professionals	Partnership between South African and Indian institutions. Requires a Masters degree or 5 years’ experience in vaccinology for admission to course.
Vaccines for Africa	University of Cape Town, South Africa	5 days	English	Annual	Immunisation programme managers, researchers and public health professionals	

## Vaccinology research

### Vaccinology literature

We identified 12 854 unique publications on vaccine-preventable diseases over the period of interest (Supplementary methods and online supplementary table 2). The total number of papers per country ranged from 0 to 2931; the number of papers per million people varied widely across countries: from 0 per million in Cape Verde to 127 per million in The Gambia (median 14 per million, IQR 6–25 per million) (online supplementary figure 4). Countries with more than 50 publications per million included in increasing order of publication rate: South Africa (53 per million, n=2931 papers, diverse research topics), Botswana (56 per million, n=122 papers, primarily tuberculosis (TB) and HIV-related research), Seychelles (65 per million, n=6 papers on dengue), Gabon (74 per million, n=128 papers, diverse research topics), Guinea Bissau (84 per million, n=155 papers, focus on non-specific vaccine effects) and The Gambia (127 per million, n=252 papers, diverse research topics).

10.1136/bmjgh-2018-001363.supp5 Supplementary data

10.1136/bmjgh-2018-001363.supp6 Supplementary data

The leading vaccine-preventable diseases in terms of research output were tuberculosis, hepatitis B, measles, human papillomavirus (HPV), dengue and cholera. There were >7 times more publications on tuberculosis than on hepatitis B in total (and >4 times more, based on the median number of published papers per country). The rank order of diseases in terms of research output varied little by geographic area. TB, hepatitis B, HPV and measles were among the top five diseases in all subregions. Polio, dengue, cholera, rotavirus and pneumococcus appeared in certain subregions but not in others (table 2).

**Table 2:** Leading vaccine-preventable diseases in terms of research output, overall and by subregion

Rank	Africa	Eastern	Western	Central	Southern
1	TB (n=9121, median=57)	TB (93%)	TB (37%)	TB (32%)	TB (98%)
2	Hepatitis B (n=1195, median=12)	Hepatitis B (6%)	Hepatitis B (11%)	Polio (14%)	Hepatitis B (9%)
3	Measles (n=715, median=8)	HPV (5%)	Polio (9%)	Hep. B (12%)	HPV (7%)
4	HPV (n=664, median=7)	Measles (5%)	Measles (7%)	Measles (9%)	Rotavirus (9%)
5	Dengue/cholera (n=547 and 524, median=z5)	Dengue/cholera (4% each)	Pneumococcus (7%)	Dengue/cholera (9% each)	Measles/pneumococcus(3% each)

N: total number of papers, median: median number of papers per country.

%: percentage of total vaccine-preventable disease publications relating to specific disease; percentages do not sum to 100% due to overlap between categories.

HPV, human papilloma virus; TB, tuberculosis.

The predominance of TB, hepatitis B and HPV publications likely reflects the association of these diseases with HIV/AIDS, which has drawn the vast majority of the region’s research funding since the 1990s. After HIV/AIDS, malaria, respiratory infections and diarrhoea are the leading causes of years of life lost in sub-Saharan Africa.^11^ However, research output for vaccine-preventable respiratory infections (pneumococcus, Hib) and diarrhoea (rotavirus) is low relative to the burden of these diseases, despite substantial investment in vaccine introduction and support for research and evaluation projects from Gavi and its partners. This trend is likely to continue for future pneumonia and diarrhoeal disease vaccines unless a concerted effort is made to fund research in this area. Conversely, polio and measles appear in the top five diseases in terms of research output in some or all subregions but now have relatively low disease burden, following major global initiatives to achieve eradication or elimination.^12 13^ Dengue and cholera are relatively rare but have garnered attention due to their recent emergence or re-emergence in the region and their potential to cause epidemics.^14 15^

### Vaccinology research sites

We compiled a list of medical and public health institutes that conduct research in vaccinology (Supplementary methods and online supplementary table 3). All subregions and most countries are home to at least one research institution, in addition to the universities we separately inventoried. However, these institutions have varying capacity (table 3). Outside of a few centres of excellence, few sites perform large-scale clinical trials or vaccine implementation research before, during or after vaccine introduction. This is problematic for vaccine advisory committees, which need data from a variety of epidemiological settings to inform policy decision-making around vaccine introduction and implementation. Areas that are particularly lacking include population-based vaccine impact studies, which require a sensitive surveillance system and a large denominator population to measure changes in disease incidence over time; implementation science studies to evaluate strategies to access difficult-to-reach populations and roll out vaccines for older age groups and modelling and cost-effectiveness analyses, which can be used to estimate disease burden, compare different immunisation schedules and evaluate the economic benefits of immunisation.

10.1136/bmjgh-2018-001363.supp7 Supplementary data

**Table 3:** Typology of vaccinology research sites

Types of site	Centres of excellence in vaccinology	Specialised vaccinology sites	Small, generalist vaccinology sites
Types of research	Cover the full range of vaccinology activities, from basic science to clinical trials and implementation research. Conduct research to international standards of quality.	Focus on 1–2 diseases and scientific disciplines of study. Generate excellent data within their area of expertise.	Have expertise in more than 1–2 diseases or disciplines. Generate data of varying quality.
External support	Have developed their research facilities through core support or large multiyear grants. Collaborate closely with one or more European or North American research institutions are often led by scientists from high-income countries.	Have developed their research facilities through network support.	–
Scale	Have reached a critical mass that enables them to sustain their capacity over time, with a regular inflow of projects to support staff and infrastructure maintenance	Are limited in the types of projects they can undertake due to their small size and narrow focus: Face scientific barriers, for example, inability to conduct multidisciplinary work or to coordinate field, clinical, laboratory and data components of projects. Face operational barriers, for example, difficulty in hiring and training large numbers of staff, challenge of managing complex grant processes, etc.	Have difficulty growing for reasons similar to those affecting specialised sites. Risk losing capacity when projects end.
Examples	KEMRI-Wellcome Trust Research Programme in Kenya; the Medical Research Council, The Gambia; the Centre for Vaccine Development, Mali; the Malawi-Liverpool-Wellcome Trust Research Programme and the Centro de Investigacao em Saude de Manhica in Mozambique, ALIVE at University of Witwatersrand	HIV research sites (International AIDS Vaccine Initiative/HIV Vaccine Trials Network sites), malaria research sites (malaria drug and vaccine trial sites), demographic surveillance sites (INDEPTH network) and laboratory science sites (Institut Pasteur International network).	

Our data also show that capacity is unevenly distributed across the region. HIV research sites, developed in response to the AIDS epidemic, are predominantly located in Southern and Eastern Africa and have international support ensuring their long-term sustainability. Malaria research sites are more geographically representative, though very few are located in Central Africa. Nigeria, DR Congo and Ethiopia have very limited research capacity. The limited capacity identified in specific subregions—particularly in Central Africa, where the risk of emerging pathogens is highest—and in the three most populous countries of the region is an impediment to conducting research representative of the epidemiological diversity of sub-Saharan Africa.

## Vaccine design and manufacturing

Africa’s limited capacity to design, manufacture and regulate vaccines is an important barrier to advancing priority vaccines through the research and development (R&D) pipeline. The need to strengthen vaccine manufacturing capacity in low-income and middle-income countries was underlined in the Global Vaccine Action Plan and reiterated in the Addis Ababa Declaration on Immunisation endorsed by African Heads of State in 2016. Local capacity would enable more rapid development of vaccines against diseases that are priorities for Africa but have a limited global market and help secure emergency supply of vaccines for Africans in the event of an outbreak or epidemic. The Institut Pasteur de Dakar (Senegal) was long the only human vaccine manufacturer in the region, producing yellow fever vaccine for UNICEF supply. In 2003, Biovac was established with support from the South African government to respond to domestic and regional vaccine needs. Biovac runs a ‘fill and finish’ facility that supplies select EPI vaccines to southern African countries and has developed and licensed a Hib conjugate vaccine technology package. It is also working to advance a pandemic influenza vaccine through the regulatory process, with support from the WHO Technology Transfer Initiative, and developing a Group B Streptococcus vaccine. In contrast to the small number of human vaccine manufacturers, approximately 20 countries have small, publicly owned or subsidised veterinary vaccine manufacturers, which rely on the Pan African Veterinary Center of the African Union for quality control and support services.

Manufacturing capacity is closely interlinked with vaccine design and regulatory capacity. Several global and local initiatives are working to strengthen these complementary areas. In order to bridge the design gap, the KEMRI-Wellcome Trust Research Programme in Kilifi, Kenya, recently created the first vaccine design laboratory in Africa, aiming to construct and evaluate human and animal vaccines of regional importance. On the regulatory side, the African Vaccine Regulatory Forum (AVAREF) was initiated by WHO AFRO in 2006 to strengthen clinical trial oversight and improve the quality and timeliness of vaccine registration processes at country level. AVAREF developed a standardised protocol format for vaccine clinical trials, provides support to national authorities for review and authorisation of trials and helps strengthen pharmacovigilance systems following vaccine introduction.

## Donor landscape

Global investment in vaccine R&D for ‘neglected diseases’ reached US$1240 million in 2014, according to our analysis of the G-finder donor database (Supplementary methods). This volume is comparable with the period 2010–2013 (excluding the US$73 million of novel funding for Ebola), but is 10% lower than total funding at its 2008–2009 peak (online supplementary figure 5). We also found that vaccine R&D for neglected diseases is primarily financed by the public sector (65%) though philanthropic organisations (21%) and private industry (14%) support a growing share of the total. Public sector funding dropped to its lowest level in 2014, whereas private industry investment peaked. Philanthropic support was close to its 8-year average and below its 2008–2009 peak (online supplementary figure 6). The US government was the leading public-sector funder, distantly followed by the European Community and the UK. The US government alone provided more than half of global funding (and >80% of public sector funding). Large decreases in US government support therefore can have a substantial impact on total R&D investment. Finally, we showed that HIV, TB and malaria received more than 80% of total vaccine R&D funding, or close to US$1 billion annually (online supplementary figure 7). After excluding Ebola, funding for ‘second tier’ neglected diseases (diarrhoeal diseases, helminth infections, kinetoplastids,^16^ dengue, bacterial pneumonia and meningitis, salmonella infections, hepatitis C, African viral haemorrhagic fevers) was fairly stable over the period 2007–2014, though investment in individual diseases showed significant year-on-year variation.

10.1136/bmjgh-2018-001363.supp8 Supplementary data

10.1136/bmjgh-2018-001363.supp9 Supplementary data

10.1136/bmjgh-2018-001363.supp10 Supplementary data

Grant data obtained from the 2015 World Report and The Bill and Melinda Gates Foundation show substantial differences in donor support across countries (online supplementary figure 8). According to these sources, 18 countries of 48 received no research support at all in 2015, while seven had more than 10 grants per 10 million people: The Gambia, Botswana, Gabon, South Africa, Kenya, Uganda and Malawi (in decreasing order). There was a 70% correlation between the number of grants a country received in 2015 and the number of publications identified through our literature search, suggesting that this metric is a reasonable proxy for research investment (despite not taking into account funding amounts) and has a substantial impact on research output.

10.1136/bmjgh-2018-001363.supp11 Supplementary data

The 2014–2015 Ebola epidemic galvanised the international community to accelerate the development of vaccines for priority emerging infections and to strengthen clinical trial infrastructure and regulatory preparedness, so that investigational vaccines could be rapidly tested in the event of a large outbreak. At the 2017 World Economic Forum, the newly established Coalition for Epidemic Preparedness and Innovation (CEPI) announced that it had received a US$460 million investment from the governments of Germany, Japan and Norway, the Wellcome Trust and the Bill and Melinda Gates Foundation and would initially focus on the development of vaccines for Nipah virus, MERS-coronovarius and Lassa fever. In parallel, the UK government committed to investing £120 million between 2016 and 2021 on the development of new vaccines for 12 priority emerging infections (three overlapping with CEPI and nine others).^17^

Overall, this analysis showed that the public sector consistently provides the majority of funding for vaccinology research, and that HIV, TB and malaria receive over 80% of investments. However, we did not include funding for basic research, as there was no way to identify projects within this category that were relevant to vaccinology from the database we used. Further, vaccine implementation, health economics, behavioural science and vaccine policy research were not reported on in the database. Finally, the data are not specific to Africa; nevertheless, since most of the ‘neglected diseases’ included in the database are prevalent in Africa, vaccine R&D focused on these diseases should be relevant to African populations regardless of individual project location.

## Conclusion

In this review and analysis of the African vaccinology landscape, we identified a large number of sites conducting vaccinology research but found that these vary widely in size and capacity and are heterogeneously distributed across the region. Training opportunities for African vaccinologists are limited. Donor funding remains overwhelmingly focused on HIV, TB and malaria vaccine development, dwarfing investment in high-burden diseases such as respiratory infections and enteric illnesses. The pipeline of new products targeting priority diseases for Africa is extensive, but rapid development is hampered by the lack of vaccine design, manufacturing and regulatory capacity in the region. There is little investment in an implementation science agenda that would increase immunisation programme efficiency and effectiveness in different settings.

These findings clearly point to specific opportunities for future donor investment in the region. In particular, offering advanced training for African vaccinologists, establishing and sustaining clinical trial and implementation research sites in underserved geographic areas, developing a multi-disciplinary approach to accelerate successful development and delivery of new vaccines against high burden pathogens beyond HIV, TB and malaria and strengthening regulatory and manufacturing capacity would contribute to the region reaching vaccine-related sustainable development goals and meeting the objectives outlined in the African Union’s Addis declaration. In order to fully benefit from donor support, sub-Saharan African countries must create the enabling environment for scientists to conduct research and identify solutions to the challenges of vaccine delivery at local level.

10.1136/bmjgh-2018-001363.supp2 Supplementary data

10.1136/bmjgh-2018-001363.supp3 Supplementary data

10.1136/bmjgh-2018-001363.supp4 Supplementary data
