# Supplementary material for: Vaccinology in sub-Saharan Africa
Source: BMJ Glob Health. 2019 Sep 20;4(5):e001363. doi: 10.1136/bmjgh-2018-001363 (PMC6768329; doi:10.1136/bmjgh-2018-001363)
Supplement: Supplementary data [file bmjgh-2018-001363supp012.pdf]

Supplementary methods

Expert interviews

We conducted in-depth interviews with 34 individuals working in vaccines and immunization at global, regional or national level. This included African, North American and European academics, WHO/HQ and WHO/AFRO staff, leaders of international NGOs, US government employees, pharmaceutical industry personnel, funding organization representatives and others. Findings from expert interviews were used to guide the research described in each of the sections below.

University search

We performed an internet-based search to obtain a complete list of universities in sub-Saharan Africa with biological science, medicine and public health programs[1]. We then specifically investigated which universities offer courses or conduct research in the fields of bacteriology, virology, immunology, epidemiology, health economics and health policy and which offer "vaccinology" training.

Literature search

We performed a PubMed search for 27 diseases, vaccines, or immunization-related terms, in 48 countries of sub-Saharan Africa, for the period January 1st 2010 through September 30th 2016 (Table 1). Titles identified in PubMed were exported to Mendeley, de-duplicated and sorted by country and disease/vaccine.

Vaccinology research site review

In order to identify and describe vaccinology research sites, we reviewed websites of research networks such as IAVI, HVTN, TB trial consortium, RTS,S-AS01 trial sites, INDEPTH demographic surveillance sites, Wellcome Trust Major Overseas Programme sites, and websites of all research institutions previously identified through the expert interviews and research network search process.

Grant data

Global donor data

The G-FINDER database reports on global investment into research and development (R&D) of new products for 35 neglected diseases, collating data from nearly 200 organizations over the period 2007-14. We used the G-FINDER search tool[2] to create a database of all R&D investments in the category of preventive vaccines, excluding basic research support. We analyzed these data to describe trends in funding over time, by funder type and disease group.

Country-specific data

The World Report website[3] includes data on all biomedical research grants active over the period 2012-2015, awarded by 12 major public and private donor organizations[4]. We downloaded data for the Africa region in 2015, selecting projects with a title or abstract that

[1] www.4icu.org/africa and individual university websites, accessed between October 25th and December 17th 2016

[2] https://gfinder.policycures.org/PublicSearchTool/,accessed 17/1/2017

3 https://worldreport.nih.gov, accessed 23/11/2016

[4] Bill and Melinda Gates Foundation, Canadian Institutes for Health and Research, US National Institutes of Health, European Commission, European Developing Countries Clinical Trials Partnership, Institut National de la Sante et de la Recherche Medicale, Institut Pasteur, Max-Planck-Gesellschaft, UK Medical Research Council, Swedish International Development Cooperation Agency, Swedish Research Council, and Wellcome Trust

included the key words “vaccine” or “immunization”. We also obtained information directly from the Bill and Melinda Gates Foundation on all grants for vaccine-related work in Africa that were active in 2015. We used these two data sources to describe the African vaccine funding landscape in 2015, by donor organization and recipient country. We did not analyze funding amounts, as these were rarely reported on.

Table 1: Disease search terms for vaccine-preventable diseases literature search

Search terms
Cholera
Dengue
Diphtheria
Haemophilus influenzae type b
Hepatitis B
Hib
HPV
Human Papilloma Virus
Immunization
Measles
Meningococcus
Neisseria meningitidis
Pertussis
Pneumococcus
Polio
Poliomyelitis
Rotavirus
Rubella
Streptococcus pneumoniae
TB
Tetanus
Tuberculosis
Typhoid
Vaccination
Vaccine Preventable Diseases
Vaccines
Yellow Fever
