# Supplementary material for: Vaccinology in sub-Saharan Africa
Source: BMJ Glob Health. 2019 Sep 20;4(5):e001363. doi: 10.1136/bmjgh-2018-001363 (PMC6768329; doi:10.1136/bmjgh-2018-001363)
Supplement: Supplementary data [file bmjgh-2018-001363supp001.pdf]

Supplementary Table 1

List of African Universities and description of training or research offerings in biological science, medicine and public health disciplines

Search completed Dec 3rd, 2016

Sources: 4icu.org/africa and university websites

University Name	Country/State	City	Website	School of Biological Science	School of Medicine	School of Public Health	Biological Science, Undergraduate	Biological Science, Masters Level	Biological Science, Doctoral Level
Universidade Católica	Angola	Luanda	http://www.ucan.edu/	No	No	Yes	No	No	No
Agostinho Neto	Angola	Luanda	http://www.agostinhone	Yes	Yes	Yes	Yes	Yes	Yes
Universidade	Angola	Luanda	http://www.unia.ao/	No	No	No	No	No	No
Universidade Técnica de	Angola	Luanda	http://www.utanga.co.ao	No	No	No	No	No	No
Universidade Metodista	Angola	Luanda	http://www.uma.co.ao/	Yes	No	Yes	Yes	No	No
Universidade Mandume	Angola	Huila	https://www.umn.ed.ao	Yes	Yes	No	Yes	No	No
Universidade Jean	Angola	Viana	http://www.unipiaget-	No	Yes	No	No	No	No
Universidade Óscar	Angola	Luanda	http://www.uor.ed.ao/	No	No	No	No	No	No
Universidade Privada de	Angola	Luanda	http://www.upra.ao/	No	Yes	No	No	No	No
Universidade Kimpa	Angola	Uíge	http://www.unikivi.com/	No	Yes	No	No	No	No
Universidade Katyavala	Angola	Benguela	http://www.ukb.ed.ao/	No	Yes	No	No	No	No
Universidade Gregório	Angola	Luanda	http://www.ugs.ed.ao/	No	No	No	No	No	No
Universidade José	Angola	Huambo	http://www.ujes-ao.org/	No	Yes	No	No	No	No
Université d'Abomey-Calavi	Benin	Atlantique	http://www.uac.bj/	Yes	Yes	No	Yes	Yes	Yes
Université d'Agriculture de Kétou	Benin	Plateau	http://www.uakbenin.org/	No	No	No	No	No	No
Université Catholique de l'Afrique de l'Ouest	Benin	Littoral	http://www.ucao-uut.tg/	No	No	No	No	No	No
Université de Parakou	Benin	Borgou	site cannot be reached	No	Yes	Yes	No	No	No

Supplementary Table 1

Université Africaine de Technologie et de Management	Benin	Cotonou	http://www.uatm-gasa.com/	No	No	No	No	No	No
Université Polytechnique d'Abomey	Benin	Zou	http://upa-benin.org/	Yes	No	No	Yes	No	No
PIGIER Benin	Benin	Littoral	http://pigier.com/	No	No	No	No	No	No
Université Polytechnique Internationale du Bénin	Benin	Littoral	http://upib-eng.com/	No	No	No	No	No	No
Université des Sciences, Arts et Techniques de Natitingou	Benin	Atakora	No website found						
Université de Lokossa	Benin	Mono	http://ul-benin.org/public/?page=ecoles&reference=instituts	No	No	No	No	No	No
Université de Porto-Novo	Benin	Oueme	http://www.upn-benin.org/	No	No	No	No	No	No
University of Botswana	Botswana	Gaborone	http://www.ub.bw/	Yes	Yes	Yes	Yes	Yes	Yes
Botswana International University of Science and Technology	Botswana	Serowe	http://www.biust.ac.bw/	Yes	No	No	Yes	Yes	Yes
Botho University	Botswana	Gaborone	http://www.bothouniversity.com/	No	No	Yes	No	No	No
Botswana Accountancy College	Botswana	Gaborone	http://www.bac.ac.bw/	No	No	No	No	No	No
Botswana College of Agriculture	Botswana	Gaborone	http://www.bca.bw/	Yes	No	No	Yes	Yes	Yes
University of Ouagadougou	Burkina Faso	Ouagadougou	http://www.univ-ouaga.bf/	Yes	Yes	No	Yes	No	No

Supplementary Table 1

Université Polytechnique de Bobo-Dioulasso	Burkina Faso	Houet	http://www.univ-bobo.bf/	Yes	Yes	No	Yes	Yes	Yes
Université Aube Nouvelle	Burkina Faso	Ouagadougou	http://www.isigburkina.org/	No	No	No	No	No	No
Université Saint Thomas d'Aquin	Burkina Faso	Ouagadougou	http://www.usta.bf/	No	Yes	No	No	No	No
Université de Koudougou	Burkina Faso	Boulkiemde	http://www.univ-koudougou.bf/	No	No	No	No	No	No
University of Ouaga II	Burkina Faso	Ouagadougou	http://www.univ-ouaga2.bf/	No	No	No	No	No	No
Université Catholique de l'Afrique de l'Ouest	Burkina Faso	Houet	http://www.ucao-uut.tg/	No	No	No	No	No	No
Université libre du Burkina	Burkina Faso	Ouagadougou	http://www.ulburkina.org/	No	No	No	No	No	No
Université Ouaga 3S	Burkina Faso	Ouagadougou	http://www.univ-ouaga3s.org/	No	No	No	No	No	No
Université Privée de Ouagadougou	Burkina Faso	Ouagadougou	http://www.univ-priveouaga.com/	No	No	No	No	No	No
University of United Popular Nations	Burkina Faso	Ouagadougou	http://www.uupn-edu.net/	No	No	No	No	No	No
Université du Burundi	Burundi	Bujumbura Mairie	http://www.ub.edu.bi/	No	Yes	No	No	No	No
Université Espoir d'Afrique	Burundi	Bujumbura	http://en.hopeafricauniversity.org/	No	Yes	Yes	No	No	No
Université Lumière de Bujumbura	Burundi	Bujumbura	http://www.ulbu.bi/	No	No	No	No	No	No
Université du Lac Tanganyika	Burundi	Bujumbura	No website found	No	No	No	No	No	No
Université de Ngozi	Burundi	Ngozi	http://univ-ngozi.bi/	No	Yes	No	No	No	No
East Africa Star University	Burundi	Bujumbura	http://www.easu-burundi.com/	No	Yes	Yes	No	No	No

Supplementary Table 1

École Normale Supérieure	Burundi	Bujumbura	http://www.ens.fr/	Yes	No	No	Yes	Yes	Yes
Université de Mwaro	Burundi	Bujumbura	http://www.universitemwaro.org/	No	No	No	No	No	No
Université de Yaoundé I	Cameroon	Yaoundé	http://www.uy1.uninet.cm/	Yes	Yes	Yes	Yes	Yes	Yes
Université de Dschang	Cameroon	Dschang	http://www.univ-dschang.org/	Yes	Yes	No	Yes	Yes	Yes
Université de Buéa	Cameroon	Buéa	http://www.ubuea.cm/	Yes	Yes	Yes	Yes	Yes	Yes
Université de Yaoundé II	Cameroon	Soa	http://www.univ-yde2.cm/uy2/	Yes	Yes	Yes	Yes	Yes	Yes
Université des Montagnes	Cameroon	Bangangté	http://www.udesmontagnes.org/	No	Yes	No	No	No	No
Université de Douala	Cameroon	Douala	http://www.univ-douala.com/	Yes	Yes	No	Yes	Yes	Yes
Université de Ngaoundéré	Cameroon	Ngaoundéré	http://www.univ-ndere.cm/	Yes	No	No	Yes	Yes	No
Université Catholique de l'Afrique Centrale	Cameroon	Yaoundé	http://www.ucac-icy.net/	No	No	No	No	No	No
Université de Bamenda	Cameroon	Bamenda	http://www.bamendauniversity.com/	Yes	Yes	Yes	Yes	No	No
Catholic University of Cameroon	Cameroon	Bamenda	http://www.catuc.org/	Yes	Yes	Yes	Yes	No	No
Université Adventiste Cosendai	Cameroon	Nanga-Eboko	http://uacosendai-edu.net/	No	No	No	No	No	No
Bamenda University of Science and Technology	Cameroon	Bamenda	http://www.bamendauniversity.com/	Yes	Yes	Yes	Yes	No	No
Université de Maroua	Cameroon	Maroua	http://www.univ-maroua.cm/	Yes	No	No	Yes	Yes	Yes
Université Protestante d'Afrique Centrale	Cameroon	Yaoundé	http://puca-edu.org/	No	No	No	No	No	No

Supplementary Table 1

Universidade Jean Piaget de Cabo Verde	Cape Verde	Praia	http://www.unipiaget.cv/	Yes	No	Yes	Yes	Yes	No
Universidade de Cabo Verde	Cape Verde	Praia	http://www.unicv.edu.cv/	Yes	Yes	Yes	Yes	Yes	Yes
Universidade de Santiago	Cape Verde	Assomada	http://us.edu.cv/	No	No	No	No	No	No
Instituto Universitário de Educação	Cape Verde	Praia	http://www.iue.edu.cv/	No	No	No	No	No	No
Instituto Superior de Ciências Económicas e Empresariais	Cape Verde	Mindelo	http://www.iscee.edu.cv/	No	No	No	No	No	No
Universidade do Mindelo	Cape Verde	Mindelo	http://www.uni-mindelo.edu.cv/	No	No	No	No	No	No
Universidade Lusófona de Cabo Verde	Cape Verde	Mindelo	http://www.ulusofona.edu.cv/	No	No	No	No	No	No
Instituto Superior de Ciências Jurídicas e Sociais	Cape Verde	Praia	http://www.iscjs.edu.cv/	No	No	No	No	No	No
Universite de Bangui	Central African Republic	Bangui	http://www.univ-bangui.org/	Yes	Yes	Yes	Yes	No	No
Université de N'Djaména	Chad	N'Djamena	http://www.univ-ndjamena.org/	Yes	Yes	Yes	Yes	No	No
University of the Comoros	Comoros	Patsy	http://www.univ-comores.km/	No	Yes	Yes	No	No	No
Kampala University	Comoros	Union des Comores	N/A	Unknown	Unknown	Unknown	Unknown	Unknown	Unknown
Université Marien Ngouabi	Congo	Brazzaville	http://www.univ-mngb.net/	Yes	Yes	No	Yes	No	No
Université de Djibouti	Djibouti	Djibouti	http://www.univ.edu.dj/	Yes	Yes	No	Yes	No	No
Université de Kinshasa	DR Congo	Kinshasa	http://www.unikin.cd/	Yes	Yes	Yes	Yes	No	No

Supplementary Table 1

Université de Lubumbashi	DR Congo	Lubumbashi	http://www.unilu.ac.cd/	No	Yes	Yes	Yes	No	No
Université Catholique du Congo	DR Congo	Kinshasa	http://ucc.ac.cd/	No	No	No	No	No	No
Université de Kisangani	DR Congo	Kisangani	http://www.unikis.ac.cd/index.php	Yes	Yes	Yes	Yes	No	No
Université Catholique de Bukavu	DR Congo	Bukavu	http://www.ucbukavu.ac.cd/	No	Yes	No	No	No	No
Université de l'Uélé	DR Congo	Isiro	N/A	No	No	No	No	No	No
Université Evangélique en Afrique	DR Congo	Bukavu	http://www.uea.ac.cd/index.php/en/	No	Yes	No	No	No	No
Université Libre des Pays des Grands Lacs	DR Congo	Goma	http://ulpgl.net/	No	No	Yes	No	No	No
Université Pedagogique Nationale	DR Congo	Kinshasa	http://www.upn-kin.cd/	Yes	No	No	No	No	No
Université Shalom de Bunia	DR Congo	Bunia	http://www.unishabunia.org/en/	No	Yes	No	No	No	No
Université Protestante du Congo	DR Congo	Kinshasa	http://www.upcrdc.org/	Yes	Yes	Yes	Yes	Yes	Yes
Université Kongo	DR Congo	Mbanza-Ngungu	http://universitekongo.org/fr/	No	Yes	No	No	No	No
Université de Bandundu	DR Congo	Kikwit	http://www.uniband.org/	No	Yes	No	No	No	No
Université Simon Kimbangu	DR Congo	Kinshasa	http://universitesk.net/	No	No	Yes	No	No	No
Université Notre-Dame du Kasayi	DR Congo	Kananga	http://www.uka-rdc.org/	Yes	Yes	Yes	Yes	No	No
Université Libre de Kinshasa	DR Congo	Kinshasa	http://ulk-rdc.org/	No	No	No	No	No	No
Université Catholique du Graben	DR Congo	Butembo	N/A	Yes	Yes	Yes	Yes	No	No

Supplementary Table 1

Université de Kikwit	DR Congo	Kikwit	http://www.unikik.net/facultes.ws	No	Yes	No	No	No	No
Université de Goma	DR Congo	Goma	http://www.unigom.org/	No	Yes	No	No	No	No
Université de Kamina	DR Congo	Kamina	http://ukamina.org/	No	Yes	Yes	No	No	No
Université Chrétienne Cardinal Malula	DR Congo	Kasa-Vubu	http://www.uccm-ispl.com/	No	Yes	Yes	No	No	No
Universidad Nacional de Guinea Ecuatorial	Equatorial Guinea	Malabo	http://www.unge.gq/unge	No	Yes	Yes	No	Yes	No
Halhale College of Business and Economics	Eritrea	Halhale	N/A	No	No	No	No	No	No
Asmara College of Nursing and Health Technology	Eritrea	Asmara	N/A	No	No	No	No	No	No
University of Asmara	Eritrea	Asmara	http://www.uoa.edu.er/	No	No	No	No	No	No
Eritrea Institute of Technology	Eritrea	Asmara	http://eit-ae.academia.edu/	Yes	No	No	Yes	No	No
Orota School of Medicine	Eritrea	Asmara	N/A	Yes	Yes	No	Yes	No	No
College of Arts and Social Sciences, Adi Keyh	Eritrea	Adi Keyh	N/A	No	No	No	No	No	No
Hamelmalo Agricultural College	Eritrea	Anseba	N/A	No	No	No	No	No	No
Addis Ababa University	Ethiopia	Addis Ababa	http://www.aau.edu.et/	Yes	Yes	Yes	Yes	No	No
Jimma University	Ethiopia	Jimma	https://www.ju.edu.et/	Yes	Yes	Yes	Yes	No	No
Hawassa University	Ethiopia	Awasa	http://www.hu.edu.et/hu/	Yes	Yes	Yes	Yes	No	N
Mekelle University	Ethiopia	Mekelle	http://www.mu.edu.et	Yes	Yes	Yes	No	No	No
Haramaya University	Ethiopia	Dire Dawa	http://www.haramaya.edu.et	Yes	Yes	Yes	Yes	Yes	No.

Supplementary Table 1

Arba Minch University	Ethiopia	Arba Minch	http://www.arbamich-univ.com	Yes	No	No	No	No	No
Bahir Dar University	Ethiopia	Bahir Dar	http://www.bdu.com	Yes	Yes	Yes	No	No	No
Adama Science and Technology University	Ethiopia	Adama	http://www.astu.edu.net	Yes	Yes	Yes	Yes	No	No
University of Gondar	Ethiopia	Gondar	http://www.uog.edu.et/	Yes	Yes	Yes	Yes	No	No
Dilla University	Ethiopia	Dilla	http://www.du.edu.et/	Yes	Yes	Yes	Yes	No	No
Ambo University	Ethiopia	Ambo	http://www.ambou.edu.et/	Yes	Yes	Yes	Yes	No	No
Wollo University	Ethiopia	Dessie	http://wollouniversity.education/	Yes	Yes	Yes	Yes	No	No
Mekelle Institute of Technology	Ethiopia	Mekelle	http://www.mitethiopia.edu.et/	Yes	No	Yes	No	No	No
Wollega University	Ethiopia	Nekemte	http://www.wollegauniversity.edu.et/	Yes	Yes	Yes	Yes	No	No
Wolkite University	Ethiopia	Welkite	http://www.wku.edu.et/	Yes	Yes	Yes	Yes	No	No
Debre Markos University	Ethiopia	Debre Markos	http://www.dmu.edu.et/	Yes	Yes	Yes	Yes	No	No
Mada Walabu University	Ethiopia	Bale Robe	http://www.mwu.edu.et/	No	Yes	Yes	No	No	No
Graduate School of Telecommunication and Information Technology	Ethiopia	Addis Ababa	http://www.gstit.edu.et/	No	No	No	No	No	No
Adigrat University	Ethiopia	Adigrat	http://www.adu.edu.et/	Yes	Yes	Yes	Yes	No	No
Debre Birhan University	Ethiopia	Debre Berhan	http://www.dbu.edu.et/	No	No	No	No	No	No
Samara University	Ethiopia	Semera	http://www.su.edu.et/	Yes	Yes	Yes	Yes	Yes	Yes
Assosa University	Ethiopia	Assosa	http://www.asu.edu.et/	Yes	No	No	Yes	No	No
Aksum University	Ethiopia	Aksum	http://www.aksumuniversity.com/	Yes	No	Yes	Yes	No	No

Supplementary Table 1

Addis Ababa Science and Technology University	Ethiopia	Addis Ababa	http://www.aastu.edu.et/	Yes	Yes	Yes	Yes	No	No
Mizan Tepi University	Ethiopia	Mizan	http://www.mtu.edu.et/	Yes	No	Yes	Yes	No	No
Woldia University	Ethiopia	Woldiya	http://wldu.edu.et/	Yes	No	Yes	Yes	No	No
Unity University	Ethiopia	Addis Ababa	http://uu.midroc-ceo.com/	No	No	No	No	No	No
Debre Tabor University	Ethiopia	Gondar	N/A	Yes	Yes	No	Yes	No	No
Wachamo University	Ethiopia	Hosaena	http://www.wcu.edu.et/	Yes	Yes	Yes	Yes	No	No
Académie Franco-Américaine de Management	Gabon	Libreville	http://aframschool.com/	No	No	No	No	No	No
Universite des Sciences de la Sante d'Owendo	Gabon	Libreville	N/A		Yes				
Institut des Techniques Avancées	Gabon	Libreville	http://www.itagabon.com/	No	No	No	No	No	No
Université des Sciences d'Informatique Appliquée	Gabon	Libreville	N/A	No	No	No	No	No	No
Université Franco-Gabonaise Saint-Exupéry	Gabon	Libreville	http://ufgse.org/	No	No	No	No	No	No
Université continentale de Libreville	Gabon	Libreville	http://www.univ-continentale.com/icspb.html	Yes	No	No	Yes	Yes	No
University of the Gambia	Gambia	Serekunda	http://unigam.academia.edu/	Yes	Yes	Yes	Yes	No	No
American International University West Africa	Gambia	Banjul	http://www.aiu.edu.gm/	No	Yes	No	No	No	No
University of Ghana	Ghana	Legon	http://www.ug.edu.gh/	Yes	Yes	Yes	Yes	No	No

Supplementary Table 1

Kwame Nkrumah University of Science and Technology	Ghana	Kumasi	https://www.knust.edu.gh/	Yes	Yes	Yes	Yes	Yes	Yes
Accra Polytechnic	Ghana	Accra	http://apoly.edu.gh/	No	No	No	No	No	No
University of Education, Winneba	Ghana	Winneba	http://www.uew.edu.gh/	Yes	No	No	Yes	Yes	Yes
University of Cape Coast	Ghana	Cape Coast	http://www.uccghanaportal.com/	Yes	Yes	No	Yes	No	No
Ashesi University College	Ghana	Berekuso	http://www.ashesi.edu.gh/	Yes	No	No	No	No	No
Ghana Institute of Management and Public Administration	Ghana	Accra	http://www.gimpa.edu.gh/	No	No	No	No	No	No
Valley View University	Ghana	Accra	https://fos.vvu.edu.gh/	No	No	No	No	No	No
Ghana Technology University College	Ghana	Accra North	http://site.gtuc.edu.gh/	No	No	No	No	No	No
University for Development Studies	Ghana	Tamale	http://www.uds.edu.gh/	Yes	Yes	No	Yes	Yes	Yes
African University College of Communication	Ghana	Accra	https://www.aucc.edu.gh/	No	No	No	No	No	No
Central University	Ghana	Tema	http://central.edu.gh/	No	No	No	No	No	No
Presbyterian University College	Ghana	Abetifi	http://www.presbyuniversity.edu.gh/site/	No	Yes	Yes	No	No	No
Regent University College of Science and Technology	Ghana	Accra	http://regent.edu.gh/	No	No	No	No	No	No
University of Mines and Technology	Ghana	Tarkwa	http://www.umat.edu.gh/	No	No	No	No	No	No
Kumasi Polytechnic	Ghana	Kumasi	https://kpoly.edu.gh/	Yes	Yes	No	Yes	No	Yes
Koforidua Polytechnic	Ghana	Koforidua	https://kpoly.edu.gh	Yes	No	No	Yes	No	No

Supplementary Table 1

Wisconsin International University College	Ghana	Accra	http://wiuc-ghana.edu.gh/	No	No	No	No	No	No
Lancaster University, Ghana	Ghana	Accra	http://www.lancaster.edu.gh/	No	No	No	No	No	No
Methodist University College	Ghana	Accra	http://www.mucg.edu.gh/	Yes	Yes	No	Yes	No	No
Catholic University College of Ghana	Ghana	Sunyani	http://www.cug.edu.gh/	No	Yes	Yes	No	No	No
Accra Institute of Technology	Ghana	Accra	http://www.ait.edu.gh/	No	No	No	No	No	No
Takoradi Polytechnic	Ghana	Takoradi	http://www.tpoly.edu.gh/	No	No	No	No	No	No
University of Professional Studies	Ghana	Accra	http://upsa.edu.gh/	No	No	No	No	No	No
Regional Maritime University	Ghana	Accra	http://www.rmu.edu.gh/	No	No	No	No	No	No
All Nations University College	Ghana	Koforidua	http://anuc.edu.gh/home/index.html	No	No	No	No	No	No
University of Health and Allied Sciences	Ghana	Ho	http://www.uhas.edu.gh/	Yes	Yes	Yes	Yes	Yes	Yes
University of Energy and Natural Resources	Ghana	Sunyani	http://uenr.edu.gh/	No	No	No	No	No	No
Pentecost University College	Ghana	Accra	http://www.pentvars.edu.gh/	No	No	No	No	No	No
BlueCrest University College	Ghana	Accra	http://www.bluecrest.edu.gh/	No	No	No	No	No	No
Ho Polytechnic	Ghana	Ho	http://www.hopoly.edu.gh/	No	No	No	No	No	No
Ghana Institute of Journalism	Ghana	Accra	http://mainsite.gij.edu.gh/	No	No	No	No	No	No

Supplementary Table 1

Christian Service University College	Ghana	Santasi-Kumasi	http://new.csuc.edu.gh/	No	No	No	No	No	No
Mountcrest University College	Ghana	Accra	http://www.mountcrestuniversity.com/	No	No	Yes	No	No	No
Garden City University College	Ghana	Kumasi	http://www.gcuc.edu.gh/	No	No	Yes	No	No	No
Cape Coast Polytechnic	Ghana	Cape Coast	http://cpoly.edu.gh/	No	No	No	No	No	No
Zenith University College	Ghana	Accra	http://zenithuniversitycollege.org/site/	No	No	No	No	No	No
Islamic University College, Ghana	Ghana	Accra	http://www.iug.edu.gh/	No	No	No	No	No	No
Knutsford University College	Ghana	Accra	http://knutsford.edu.gh/	No	No	No	No	No	No
Ghana Christian University College	Ghana	Accra	http://www.ghanacu.org/	No	No	Yes	No	No	No
Kings University College	Ghana	Accra	http://www.kuc.edu.gh/	No	No	No	No	No	No
Ghana Baptist University College	Ghana	Abuakwa-Kumasi	http://gbuc.edu.gh/	No	No	No	No	No	No
Pan African Christian University College	Ghana	Accra	http://www.pacuc.edu.gh/	No	No	No	No	No	No
Marshalls University College	Ghana	Accra	http://www.marshalls.edu.gh/	No	No	No	No	No	No
Maranatha University College	Ghana	Accra North	http://maranathauniversity.org/	No	No	No	No	No	No
Radford University College	Ghana	Accra	http://www.radforduc.edu.gh/	Yes	No	Yes	Yes	No	No
Evangelical Presbyterian University College	Ghana	Ho	http://www.epuconline.com/ADMISSIONBROCHURE.pdf	No	No	No	No	No	No
University College of Management Studies	Ghana	Accra	http://www.ucoms.edu.gh/	No	No	No	No	No	No

Supplementary Table 1

KAAF University College	Ghana	Mallam	https://www.kaafuniversitycollege.edu.gh	No	No	No	No	No	No
Tamale Polytechnic	Ghana	Tamale	http://tamalepoly.edu.gh/	No	No	No	No	No	No
Spiritan University College	Ghana	Ejisu	http://www.spiritanuc.edu.gh/	No	No	No	No	No	No
Sunyani Polytechnic	Ghana	Sunyani	http://www.spoly.edu.gh/en/	No	Yes	No	No	No	No
University College of Agriculture and Environmental Studies	Ghana	Bunso	http://ucaes.edu.gh/	No	No	No	No	No	No
Anglican University College of Technology	Ghana	Teshie	http://www.angutech.edu.gh/	No	Yes	No	No	No	No
Bolgatanga Polytechnic	Ghana	Bolgatanga	http://www.bpoly.edu.gh/	No	No	No	No	No	No
Dominion University College	Ghana	Accra	http://www.duc.edu.gh/	No	No	No	No	No	No
Ghana Institute of Languages	Ghana	Accra	http://www.gil.edu.gh/	No	No	No	No	No	No
Catholic Institute of Business and Technology	Ghana	Accra North	http://www.cibtghana.net/	No	No	No	No	No	No
West End University College	Ghana	Accra	http://www.weuc.edu.gh/	No	No	No	No	No	No
Christ Apostolic University College	Ghana	Kumasi	http://www.cauc.edu.gh/	No	No	No	No	No	No
Advanced Business College	Ghana	Accra	http://www.advancedbusinesscollege.com/	No	No	No	No	No	No
Jayee University College	Ghana	Accra	http://www.juc.edu.gh/	No	No	No	No	No	No
Data Link Institute	Ghana	Tema	http://www.datalink.edu.gh/	No	No	No	No	No	No

Supplementary Table 1

Université Gamal Abdel Nasser de Conakry	Guinea	Conakry		Yes	Yes	No	Yes	Yes	No
Université Kofi Annan de Guinée	Guinea	Conakry	http://www.univ-kag.org/	No	Yes	No	No	No	No
Universidade Colinas de Boe (UCB)	Guinea Bissau	Bissau	N/A	Unknown	Unknown	Unknown	Unknown	Unknown	Unknown
Universidade Lusófona Guiné / Universidade Amílcar Cabral (UAC)- Guinea Bissau	Guinea Bissau	Bissau	http://ulg.grupolusofona.pt/	No	No	No	No	No	No
UNIPIAGET- Universidade Jean Piaget Guine-Bissau	Guinea Bissau	Bissau	http://guine-bissau.unipiaget.org/	No	Yes	No	No	No	No
Pentecostal Life University	Ivory Coast	Lilongwe	http://www.plu.mw/	No	No	No	No	No	No
Institut National Polytechnique Félix Houphouët-Boigny	Ivory Coast	Yamoussoukro	http://www.inphb.edu.ci/	No	No	No	No	No	No
Université Félix Houphouët-Boigny	Ivory Coast	Abidjan	http://univ-fhb.edu.ci/	Yes	Yes	No	Yes	No	No
École Normale Supérieure d'Abidjan	Ivory Coast	Abidjan	http://ensabidjan.org/	Yes	No	No	Yes	No	No
Institut National Supérieur des Arts et de l'Action Culturelle	Ivory Coast	Abidjan	N/A	No	No	No	No	No	No
École Supérieure Africaine des Technologies de l'Information et de la Communication	Ivory Coast	Abidjan	http://www.esatic.ci/	No	Yes	No	No	No	No

Supplementary Table 1

Université Nangui Abrogoua	Ivory Coast	Abidjan	http://www.univ-na.edu.ci/	No	No	No	No	No	No
Institut des Sciences et Techniques de la Communication	Ivory Coast	Abidjan	N/A	No	No	No	No	No	No
École Nationale d'Administration	Ivory Coast	Abidjan	http://www.ena.ci/	No	No	No	No	No	No
Université Péléforo-Gbon-Coulibaly	Ivory Coast	Korhogo ...	http://www.univ-pgc.edu.ci/	Yes	Yes	No	Yes	No	No
École Nationale Supérieure de Statistique et d'Economie Appliquée	Ivory Coast	Abidjan	http://www.ensea.ed.ci/	No	No	No	No	No	No
Université Jean Lorougnon Guédé	Kenya	Daloa	http://www.ujlog.ci/	Yes	No	No	No	No	No
University of Nairobi	Kenya	Nairobi	http://www.uonbi.ac.ke/	Yes	Yes	Yes	Yes	Yes	Yes
Egerton University	Kenya	Njoro	http://www.egerton.ac.ke/	Yes	Yes	Yes	Yes	Yes	Yes
Kenyatta University	Kenya	Nairobi	http://www.ku.ac.ke/	Yes	Yes	Yes	No	Yes	No
Moi University	Kenya	Eldoret	https://www.mu.ac.ke/	Yes	Yes	Yes	Yes	Yes	Yes
Strathmore University	Kenya	Nairobi	http://www.strathmore.edu/en/	No	No	No	No	No	No
Jomo Kenyatta University of Agriculture and Technology	Kenya	Nairobi	http://www.jkuat.ac.ke/	Yes	Yes	Yes	Yes	Yes	Yes
United States International University	Kenya	Nairobi	http://www.usiu.ac.ke/	No	No	No	No	No	No
Mount Kenya University	Kenya	Thika	http://www.mku.ac.ke/	Yes	Yes	Yes	Yes	Yes	Yes

Supplementary Table 1

Catholic University of Eastern Africa	Kenya	Nairobi	http://www.cuea.edu/	No	No	No	No	No	No
University of Eastern Africa, Baraton	Kenya	Eldoret	http://ueab.ac.ke/	Yes	Yes	Yes	Yes	Yes	Yes
Maseno University	Kenya	Maseno	http://maseno.ac.ke/	Yes	Yes	Yes	Yes	Yes	Yes
Kenya Methodist University	Kenya	Meru	http://www.kemu.ac.ke/	No	Yes	Yes	No	No	No
Daystar University	Kenya	Nairobi	http://www.daystar.ac.ke/	Yes	No	No	Yes	No	No
Kabarak University	Kenya	Nakuru	http://www.kabarak.ac.ke/	Yes	Yes	Yes	Yes	Yes	Yes
Zetech University	Kenya	Nairobi	http://zetech.ac.ke/	No	No	No	No	No	No
Africa Nazarene University	Kenya	Nairobi	http://www.anu.ac.ke/	No	No	No	No	No	No
Technical University of Kenya	Kenya	Nairobi	http://tukenya.ac.ke/	Yes	No	Yes	Yes	Yes	No
KCA University	Kenya	Nairobi	http://www.kca.ac.ke/	No	No	No	No	No	No
Dedan Kimathi University of Technology	Kenya	Nyeri	http://www.dkut.ac.ke/	No	No	No	No	No	No
Masinde Muliro University of Science and Technology	Kenya	Kakamega	http://www.mmust.ac.ke/	Yes	Yes	Yes	Yes	No	No
Technical University of Mombasa	Kenya	Mombasa	http://www.tum.ac.ke/	Yes	Yes	Yes	Yes	Yes	Yes
Chuka University	Kenya	Chuka	http://chuka.ac.ke/	Yes	No	No	Yes	No	No
St. Paul's University	Kenya	Limuru	http://spu.ac.ke/	No	No	No	No	No	No
Pwani University	Kenya	Kilifi	http://www.pu.ac.ke/	Yes	No	Yes	Yes	Yes	Yes
Multimedia University of Kenya	Kenya	Nairobi	http://www.mmu.ac.ke/	No	No	No	No	No	No

Supplementary Table 1

University of Eldoret	Kenya	Eldoret	http://www.uoeld.ac.ke/karibu/	Yes	No	No	Yes	Yes	Yes
Africa International University	Kenya	Nairobi	http://www.aiu.ac.ke/	No	No	No	No	No	No
Karatina University	Kenya	Karatina	https://www.karu.ac.ke/	Yes	No	No	Yes	Yes	Yes
South Eastern Kenya University	Kenya	Kitui	http://www.seku.ac.ke/	No	No	Yes	No	No	No
Jaramogi Oginga Odinga University of Science and Technology	Kenya	Bondo	http://jooust.ac.ke/	Yes	No	Yes	Yes	Yes	Yes
Great Lakes University of Kisumu	Kenya	Kisumu	http://www.gluk.ac.ke/	No	Yes	Yes	No	No	No
Laikipia University	Kenya	Nyahururu	http://www.laikipia.ac.ke/	Yes	No	No	Yes	Yes	Yes
Meru University of Science and Technology	Kenya	Meru	http://www.must.ac.ke/	Yes	Yes	Yes	Yes	Yes	Yes
Kibabii University	Kenya	Bungoma	http://www.kibabiiuniversity.ac.ke/	Yes	No	No	Yes	Yes	Yes
Maasai Mara University	Kenya	Narok	http://www.mmarau.ac.ke/	Yes	No	No	Yes	Yes	Yes
Kisii University	Kenya	Kisii	http://www.kisiiuniversity.ac.ke/	Yes	Yes	Yes	Yes	Yes	Yes
Adventist University of Africa	Kenya	Nairobi	http://aua.ac.ke/	No	No	Yes	No	No	No
Pan Africa Christian University	Kenya	Nairobi	http://www.pacuniversity.ac.ke/	No	No	No	No	No	No
Management University of Africa	Kenya	Nairobi	http://www.mua.ac.ke/	No	No	No	No	No	No
Kiriri Women's University of Science and Technology	Kenya	Nairobi	http://www.kwust.ac.ke/	No	No	No	No	No	No

Supplementary Table 1

Riara University	Kenya	Nairobi	http://www.riarauniversity.ac.ke/	No	No	No	No	No	No
University of Kabianga	Kenya	Kericho	http://kabianga.ac.ke/main/	No	No	No	No	No	No
Inoorero University	Kenya	Nairobi	http://www.iu.ac.ke/	No	No	No	No	No	No
Scott Christian University	Kenya	Machakos	http://www.scott.ac.ke/	No	No	No	No	No	No
Gretsa University	Kenya	Thika	http://www.gretsauniversity.ac.ke/	Yes	No	Yes	Yes	No	No
The Presbyterian University of East Africa	Kenya	Kikuyu	http://www.puea.ac.ke/	No	Yes	No	No	No	No
Umma University	Kenya	Kajiado	https://www.umma.ac.ke/	No	Yes	No	No	No	No
Kenya Highlands Evangelical University	Kenya	Kericho	http://www.kheu.ac.ke/	No	No	No	No	No	No
The East African University	Kenya	Nairobi	http://www.teau.ac.ke/	No	No	Yes	No	No	No
Pioneer International University	Kenya	Nairobi	http://www.piu.ac.ke/	No	No	No	No	No	No
Lukenya University	Lesotho	Athi River	http://www.lukenyauniversity.ac.ke/	No	No	No	No	No	No
National University of Lesotho	Lesotho	Maseru	http://www.nul.ls/	No	No	No	No	No	No
Lesotho College of Education	Liberia	Maseru	http://www.lce.ac.ls/	No	No	No	No	No	No
Cuttington University	Liberia	Monrovia	http://cuttingtonuniversity.edu.lr/	Yes	Yes	Yes	Yes	No	No
AME Zion University	Liberia	Monrovia	http://www.ame.edu.lr/	No	No	No	No	No	No
United Methodist University	Liberia	Monrovia	http://umu.edu.lr/	No	Yes	No	No	No	No
University of Liberia	Madagascar	Monrovia	http://www.ul.edu.lr/	No	Yes	Yes	No	No	No

Supplementary Table 1

Universite d' Antananarivo	Madagascar	Antananarivo	http://www.univ-antananarivo.mg/	Yes	Yes	No	Yes	Yes	Yes
Universite de Mahajanga	Madagascar	Mahajanga	http://www.univ-mahajanga.edu.mg/	Yes	Yes	Yes	Yes	Yes	Yes
Universite d' Antsiranana	Madagascar	Antsiranana	http://www.univ-antsiranana.edu.mg/	No	No	No	No	No	No
Universite de Toamasina	Malawi	Toamasina	http://www.univ-toamasina.mg/	No	No	No	No	No	No
University of Malawi	Malawi	Zomba	http://www.medcol.mw/	Yes	Yes	Yes	Yes	Yes	Yes
Malawi University of Science and Technology	Malawi	Limbe	http://www.must.ac.mw/	Yes	No	No	Yes	Yes	Yes
Mzuzu University	Malawi	Mzuzu	http://www.mzuni.ac.mw/	Yes	No	No	Yes	Yes	No
Malawi Adventist University	Malawi	Ntcheu	http://mau.ac.mw/	Yes	No	No	Yes	No	No
Malawi Assemblies of God University	Malawi	Lilongwe	http://www.magu.ac.mw/	No	No	No	No	No	No
Blantyre International University	Malawi	Lilongwe	http://www.cybertechmw.com/biu/study-programmes/	No	No	No	No	No	No
Nkhoma University	Malawi	Lilongwe	http://www.nkhoma.ac.mw/views/courses.html	No	No	No	No	No	No
The Catholic University of Malawi	Malawi	Limbe	http://www.cunima.ac.mw/	Yes	No	No	Yes	No	No
Lilongwe University of Agriculture and Natural Resources	Malawi	Lilongwe	http://luanar.academia.edu/	Yes	No	No	Yes	Yes	Yes
University of Livingstonia	Malawi	Livingstonia	http://mwluv.com/list-courses-offered-university-livingstonia-unilia/	Yes	Yes	Yes	Yes	No	No

Supplementary Table 1

St. John The Baptist University	Malawi	Lilongwe	http://dmisjbu.edu.mw/	No	No	No	No	No	No
Riverton University	Malawi	Lilongwe	http://rivertonmw.com/	No	No	No	No	No	No
Skyway University	Malawi	Lilongwe	http://www.skywayuniversity.net/	No	No	Yes	No	No	No
Daeyang University	Malawi	Lilongwe	http://www.university.daeyangmission.org/index.php/college-of-nursing	No	No	No	No	No	No
École Nationale d'Ingénieurs Abderhame Baba Touré	Mali	Bamako	http://www.eni-abt.com/	No	No	No	No	No	No
Institut Polytechnique Rural de Formation et de Recherches Appliquées de Katibougou	Mali	Katibougou	http://www.ipr-ifra.org/	No	No	No	No	No	No
Université des sciences, des techniques et des technologies de Bamako	Mali	Bamako	http://www.fst-usttb-edu.ml/	Yes	Yes	No	Yes	No	No
Université des sciences juridiques et politiques de Bamako	Mali	Bamako	http://www.usjpb.edu.ml/	No	No	No	No	No	No
Université des lettres et	Mali	Bamako	http://www.ulshb.edu.ml	No	No	No	No	No	No
École Normale	Mali	Bamako	http://enetp.edu.ml/	No	No	No	No	No	No
Université de	Mauritania	Nouakchott	http://www.univ-nkc.mr/	Yes	Yes	Yes	Yes	No	No
Université Libanaise	Mauritania	Nouakchott	http://www.liu.edu.lb/	Yes	No	No	Yes	No	No
École Nationale	Mauritania	Nouakchott	http://enajm.mr/	No	No	No	No	No	No
University of Mauritius	Mauritius	Reduit	http://www.uom.ac.mu/	Yes	Yes	Yes	Yes	Yes	No
Mauritius Institute of	Mauritius	Reduit	http://www.mie.ac.mu/	No	No	No	No	No	No

Supplementary Table 1

University of	Mauritius	Pointes-Aux-Sablas	http://www.utm.ac.mu/	Yes	Yes	Yes	Yes	Yes	Yes
Mahatma Gandhi	Mauritius	Moka	http://mgirti.org/	No	No	No	No	No	No
Universidade Eduardo	Mozambique	Maputo	http://www.uem.mz/	Yes	Yes	Yes	Yes	Yes	No
Universidade Católica	Mozambique	Beira	http://www.ucm.ac.mz/c	Yes	Yes	No	Yes	No	No
Universidade	Mozambique	Maputo	http://www.apolitecnica.	No	Yes	Yes	No	No	No
University of Namibia	Namibia	Windhoek	http://www.unam.edu.n	No	Yes	Yes	No	No	No
Namibia University of	Namibia	Windhoek	http://www.nust.na/	Yes	Yes	No	Yes	Yes	No
The International	Namibia	Windhoek	http://www.ium.edu.na/	Yes	No	No	Yes	No	No
Université Abdou	Niger	Niamey	http://uam.refer.ne/	Yes	Yes	No	Yes	Yes	No
University of Ibadan	Nigeria	Ibadan	http://www.ui.edu.ng/	Yes	Yes	Yes	Yes	Yes	Yes
University of Lagos	Nigeria	Lagos	http://www.unilag.edu.ng/	Yes	Yes	Yes	Yes	Yes	Yes
Covenant University	Nigeria	Ota	http://covenantuniversity.edu.ng/	Yes	No	No	Yes	Yes	Yes
Obafemi Awo Iowo University	Nigeria	Osun	http://www.oauife.edu.ng/	Yes	Yes	Yes	Yes	Yes	Yes
University of Ilorin	Nigeria	Kwara	https://www.unilorin.edu.ng/	Yes	Yes	Yes	Yes	Yes	Yes
University of Nigeria	Nigeria	Enugu	http://www.unn.edu.ng/	Yes	Yes	Yes	Yes	Yes	Yes
Federal University of Technology	Nigeria	Imo	http://www.futa.edu.ng/futacms/	Yes	No	Yes	Yes	No	No
University of Benin	Nigeria	Edu	http://lifesci.uniben.edu/#	Yes	Yes	Yes	Yes	Yes	Yes
Lagos State University	Nigeria	Lagos	http://www.lasu.edu.ng/	Yes	Yes	No	Yes	No	No
Michael Okpara University of Agriculture	Nigeria	Abia	http://mouau.edu.ng/	Yes	No	No	No	No	No
Federal University, Oye-Ekiti	Nigeria	Oye	http://fuoye.edu.ng/en/	Yes	No	No	Yes	No	No

Supplementary Table 1

Babcock University	Nigeria	Ogun	http://www.babcock.edu.ng/	Yes	Yes	Yes	Yes	Yes	No
Ahmadu Bello University	Nigeria	Kaduna	https://abu.edu.ng/	Yes	Yes	Yes	Yes	Yes	Yes
University of Agriculture, Abeokuta	Nigeria	Ogun	http://www.unaab.edu.ng/	Yes	No	No	Yes	Yes	Yes
Kwara State University	Nigeria	Kwara	http://www.kwasu.edu.ng/	Yes	No	No	Yes	No	No
Landmark University	Nigeria	Omu-Aran	http://lmu.edu.ng/	Yes	No	No	Yes	No	No
University of Port Harcourt	Nigeria	Rivers	http://www.uniport.edu.ng/	Yes	Yes	No	Yes	Yes	Yes
Afe Babalola University	Nigeria	Ado-Ekiti	http://abuad.edu.ng/	Yes	No	Yes	Yes	Yes	Yes
Bayero University Kano	Nigeria	Kano	http://www.buk.edu.ng/	Yes	Yes	Yes	Yes	Yes	No
Ekiti State University, Ado Ekiti	Nigeria	Ado Ekiti	http://www.eksu.edu.ng/	Yes	Yes	Yes	Yes	Yes	No
Olabisi Onabanj University	Nigeria	Ogun	http://www.oouagoiwoye.edu.ng/	Yes	Yes	Yes	Yes	No	No
Federal University of Technology	Nigeria	Akure	http://fuoye.edu.ng/en/	Yes	No	No	Yes	No	No
University of Jos	Nigeria	Plateau	http://www.unijos.edu.ng/	Yes	Yes	No	Yes	Yes	Yes
Federal University of Technology, Minna	Nigeria	Minna	https://www.futminna.edu.ng/	Yes	Yes	No	Yes	No	No
Tai Solarin University of Education	Nigeria	Ogun	https://tasued.edu.ng/	Yes	No	Yes	Yes	Yes	No
Enugu State University of Science &	Nigeria	Enugu	http://www.esut.edu.ng/	Yes	Yes	No	Yes	No	No
Veritas University	Nigeria	Federal Capital Territory	http://www.veritas.edu.ng/	Yes	No	Yes	Yes	No	No
Federal University Dutsin-Ma	Nigeria	Katsina	http://fudutsinma.edu.ng/	Yes	No	No	Yes	No	No

Supplementary Table 1

Ladoke Akintola University of Technology	Nigeria	Oyo	http://lautech.edu.ng/	Yes	Yes	Yes	Yes	Yes	Yes
University of Uyo	Nigeria	Uyo	http://www.uniuyo.edu.ng/	Yes	Yes	Yes	Yes	Yes	Yes
University of Abuja	Nigeria	Federal Capital Territory	https://www.uniabuja.edu.ng/	Yes	Yes	Yes	Yes	Yes	Yes
Pan African University	Nigeria	Lagos	http://www.pau.edu.ng/	No	No	No	No	No	No
Nnamdi Azikiwe University	Nigeria	Anambra	http://www.unizik.edu.ng/	Yes	Yes	No	Yes	Yes	Yes
American University of Nigeria	Nigeria	Adamawa	http://www.aun.edu.ng/	Yes	No	No	Yes	Yes	Yes
Adekunle Ajasin University	Nigeria	Ondo	https://aaua.edu.ng/b/	Yes	Yes	Yes	Yes	No	No
Rivers State University of Science and Technology	Nigeria	Rivers	http://www.ust.edu.ng/	Yes	Yes	No	Yes	Yes	Yes
Bowen University	Nigeria	Iwo	http://bowenuniversity.edu.ng/	Yes	Yes	No	Yes	Yes	Yes
Delta State University, Abraka	Nigeria	Abraka	http://www.delsu.edu.ng/	Yes	Yes	No	Yes	Yes	Yes
African University Of Science And Technology	Nigeria	Abuja	https://aust.edu.ng/	No	No	No	No	No	No
Redeemer's University	Nigeria	Mpwe	http://www.run.edu.ng/	Yes	Yes	No	Yes	Yes	Yes
Chukwuemeka Odumegwu Ojukwu University	Nigeria	Anambra	http://coou.edu.ng/	Yes	No	Yes	Yes	Yes	Yes
Osun State University	Nigeria	Oshogbo	http://www.uniosun.edu.ng/	Yes	Yes	Yes	Yes	No	No
Ibrahim Badamasi Babangida University	Nigeria	Niger	http://www.ibbu.edu.ng/	Yes	Yes	No	Yes	No	No

Supplementary Table 1

Usmanu Danfodio University	Nigeria	Sokoto	http://www.udusok.edu.ng/	Yes	Yes	Yes	Yes	Yes	Yes
University of Calabar	Nigeria	Cross River	http://www.unical.edu.ng/	Yes	Yes	Yes	Yes	Yes	Yes
Ebonyi State University	Nigeria	Ebonyi	http://ebsu-edu.net/	Yes	Yes	No	Yes	Yes	Yes
Baze University	Nigeria	Federal Capital Territory	http://bazeuniversity.edu.ng/	Yes	Yes	No	Yes	No	No
Abubakar Tafawa Balewa University	Nigeria	Bauchi	http://bazeuniversity.edu.ng/	Yes	No	Yes	Yes	No	No
Kaduna State University	Nigeria	Kaduna	http://www.kasuportal.com/	Yes	Yes	No	Yes	Yes	Yes
University of Agriculture, Makurdi	Nigeria	Makurdi	http://uam.edu.ng/	Yes	Yes	No	Yes	Yes	Yes
Federal University of Petroleum Resources	Nigeria	Delta	http://www.fupre.edu.ng/s/	Yes	No	No	Yes	Yes	Yes
Belle University of Technology	Nigeria	Ogun	http://www.bellsuniversity.edu.ng/	Yes	No	No	Yes	No	No
University of Maiduguri	Nigeria	Borno	http://www.unimaid.edu.ng/	Yes	Yes	Yes	Yes	Yes	Yes
Lead City University	Nigeria	Oyo	http://www.lcu.edu.ng/	Yes	No	Yes	Yes	No	No
Narasawa State University	Nigeria	Narasawa	http://nsuk.edu.ng/	Yes	No	Yes	Yes	Yes	Yes
Imo State University	Nigeria	Imo	http://imsu.edu.ng/site/	Yes	Yes	Yes	Yes	Yes	Yes
Ambrose Alli University	Nigeria	Edo	https://www.aauekpoma.edu.ng	Yes	Yes	Yes	Yes	Yes	Yes
Bingham University	Nigeria	Nasawasa	http://www.binghamuni.edu.ng/	Yes	Yes	No	Yes	No	No
Niger Delta University	Nigeria	Bayelsa	http://www.ndu.edu.ng/	Yes	Yes	Yes	Yes	Yes	Yes
Ajayi Crowther University	Nigeria	Oyo	http://www.acu.edu.ng/	Yes	No	No	Yes	Yes	Yes

Supplementary Table 1

Federal University, Ndufu-Alike	Nigeria	Ndufu-Alike	http://www.funai.edu.ng/	Yes	Yes	Yes	Yes	Yes	Yes
Modibbo Adama University of Technology	Nigeria	Adamawa	http://mautech.edu.ng/	Yes	Yes	No	Yes	Yes	Yes
Abia State University	Nigeria	Abia	http://www.absu.edu.ng	Yes	Yes	Yes	Yes	No	No
Benue State University	Nigeria	Benue	https://www.bsum.edu.ng	Yes	No	No	Yes	Yes	Yes
Igbinedion University, Okada	Nigeria	Edo	http://www.iuokada.edu.ng/	Yes	Yes	No	Yes	Yes	Yes
Umaru Musa Yar'Adua University	Nigeria	Katsina	http://www.umyu.edu.ng/	Yes	Yes	No	Yes	Yes	Yes
Paul University	Nigeria	Anambra	http://www.pauluniversity.edu.ng/	Yes	No	No	Yes	No	No
Nigerian Turkish Nile University	Nigeria	Federal Capital Territory	http://www.ntnu.edu.ng/	Yes	Yes	No	Yes	Yes	Yes
Madonna University	Nigeria	Anambra	http://www.madonnauniversity.edu.ng/	Yes	Yes	Yes	Yes	Yes	Yes
Benson Idahosa University	Nigeria	Edo	http://www.biu.edu.ng/	Yes	Yes	Yes	Yes	Yes	Yes
Crawford University	Nigeria	Ogun	http://www.crawforduniversity.edu.ng/	Yes	No	No	Yes	No	No
Achievers University	Nigeria	Owo	http://achievers.edu.ng/	Yes	No	No	Yes	Yes	Yes
Caleb University	Nigeria	Lagos	http://calebuniversity.edu.ng	Yes	No	No	Yes	Yes	Yes
Joseph Ayo Babalola University	Nigeria	Osun	http://www.jabu.edu.ng/	Yes	No	No	Yes	Yes	Yes
Ondo State University of Science and Technology	Nigeria	Ondo	http://www.osustech.edu.ng/	No	No	No	No	No	No

Supplementary Table 1

Federal University, Otuoke	Nigeria	Otuoke	http://www.fuotuoke.edu.ng/	Yes	No	No	Yes	Yes	Yes
Elizade University	Nigeria	Ondo	http://www.elizadeuniversity.edu.ng/	Yes	Yes	Yes	Yes	No	No
Oduduwa University	Nigeria	Osun	http://oduduwauniversity.edu.ng/	Yes	Yes	Yes	Yes	No	No
Caritas University	Nigeria	Enugu	http://www.caritasuni.edu.ng/	No	No	No	No	No	No
Kano University of Science and Technology	Nigeria	Kano	N/A	Yes	No	No	Yes	No	No
Kogi State University	Nigeria	Kogi	http://www.ksu.edu.ng/	Yes	Yes	No	Yes	No	No
Cross River University of Technology	Nigeria	Cross River	http://crutech.edu.ng/	Yes	No	No	Yes	No	No
Federal University, Lokoja	Nigeria	Lokoja	http://www.fulokoja.edu.ng/	Yes	No	No	Yes	No	No
Fountain University	Nigeria	Osogbo	http://www.fountainuniversity.edu.ng/	Yes	No	No	Yes	No	No
Federal University, Dutse	Nigeria	Jigawa	http://fud.edu.ng/	Yes	Yes	No	Yes	Yes	No
Akwa Ibom State University	Nigeria	Akwa Ibom	https://www.aksu.edu.ng/	Yes	No	No	Yes	Yes	Yes
Al-Hukmah University	Nigeria	Kwara	http://alhikmah.edu.ng/	Yes	No	No	Yes	No	No
Adeleke University	Nigeria	Osun	http://adelekeuniversity.edu.ng/	Yes	No	No	Yes	Yes	Yes
Wellspring University	Nigeria	Edo	N/A	Yes	No	No	Yes	No	No
Salem University	Nigeria	Kogi	http://salemuniversity.edu.ng/	Yes	No	No	No	No	No
Federal University, Lafia	Nigeria	Nasawasa	http://www.fulafia.edu.ng/	Yes	No	No	Yes	Yes	Yes
University of Mkar	Nigeria	Benue	http://www.unimkar.edu.ng/	Yes	No	No	Yes	No	No

Supplementary Table 1

Obong University	Nigeria	Akwa Ibom	http://www.obonguniversity.net/	Yes	No	No	Yes	No	No
Renaissance University	Nigeria	Enugu	http://www.rnu.edu.ng/	Yes	No	No	Yes	No	No
Yobe State University	Nigeria	Yobe	http://www.ysu.edu.ng/	Yes	No	No	Yes	No	No
Federal University, Wukari	Nigeria	Taraba	http://fuwukari.edu.ng/	Yes	No	No	Yes	Yes	Yes
Gombe State University	Nigeria	Gombe	http://gsu.edu.ng/	Yes	Yes	No	Yes	Yes	No
Kwararafa University Wukari	Nigeria	Taraba	http://www.kwararafauniversity.edu.ng/home/index.php/en/	Yes	No	No	Yes	Yes	Yes
Sokoto State University	Nigeria	Sokoto	http://www.ssu.edu.ng/	Yes	No	No	Yes	Yes	No
Tansian University	Nigeria	Anambra	http://www.tansianuniversity.edu.ng/	Yes	No	No	Yes	No	No
Taraba State University	Nigeria	Taraba	http://www.tsuniversity.edu.ng/	Yes	No	No	No	No	No
Godfreye Okoye University	Nigeria	Enugu	http://gouni.edu.ng/	Yes	Yes	No	Yes	Yes	Yes
Kebbi State University of Science and Technology	Nigeria	Kebbi	http://www.ksusta.edu.ng/	Yes	No	No	Yes	No	No
Wesley University of Science and Technology	Nigeria	Ondo City	http://www.sau.edu.ng/	Yes	Yes	No	Yes	Yes	Yes
Samuel Adegboyega University	Nigeria	Edo	http://www.sau.edu.ng/	Yes	Yes	No	Yes	Yes	Yes
Al Qalam University, Katsina	Nigeria	Katsina	http://www.auk.edu.ng	Yes	No	No	Yes	Yes	Yes
Rhema University	Nigeria	Rivers	http://www.rhemauniversity.edu.ng/	Yes	Yes	Yes	Yes	No	No
Université de la Reunion	Reunion	Saint Denis	http://www.univ-reunion.fr/	Yes	No	No	Yes	No	No
University of Rwanda	Rwanda	Kigali City	http://www.ce.ur.ac.rw/	Yes	Yes	Yes	Yes	Yes	Yes

Supplementary Table 1

Kibogora Polytechnic	Rwanda	Western Province	http://www.kp.ac.rw/	No	No	No	No	No	No
Université Libre de Kigali	Rwanda	Kigali City	http://ulk-kigali.net/	No	No	No	No	No	No
Adventist University of Central Africa	Rwanda	Kigali City	http://www.auca.ac.rw	No	Yes	No	No	No	No
Institut d'Enséignement Supérieur de Ruhengeri	Rwanda	Northern Province	http://www.auca.ac.rw/	No	No	No	No	No	No
University of Lay Adventists of Kigali	Rwanda	Kigali City	http://www.unilak.ac.rw /	Yes	No	No	Yes	Yes	Yes
Kigali Institute Of Management	Rwanda	Kigali City	http://kimuniversity.ac.r w/	No	No	No	No	No	No
University of Kigali	Rwanda	Kigali City	http://uok.ac.rw/	No	No	No	No	No	No
Institute of Legal Practice &	Rwanda	Southern Province	http://ilpd.ac.rw/home/	No	No	No	No	No	No
Université Catholique de Kabgayi	Rwanda	Southern Province	http://uck.ac.rw/	No	No	No	No	No	No
Protestant Institute of Arts and Social Sciences	Rwanda	Southern Province	http://www.piass.ac.rw/	No	No	No	No	No	No
Institut Polytechnique de Byumba	Rwanda	Northern Province	http://www.byumbapoly technic.com/	Yes	No	No	Yes	No	No
University of Kibungo	Rwanda	Eastern Province	http://www.unik.ac.rw/	No	No	No	No	No	No
Catholic University of Rwanda	Rwanda	Southern Province	http://www.cur.ac.rw/	No	No	Yes	No	No	No
Public University of São Tomé and Príncipe	Sao Tome and Principe		N/A	Unknown	Unknown	Unknown	Unknown	Unknown	Unknown
Lusíada University of Sao Tome and Principe	Sao Tome and Principe	Porto	N/A	Unknown	Unknown	Unknown	Unknown	Unknown	Unknown
University Institute of Accounting , Management and Computing	Sao Tome and Principe	Avenue Amilcar Cabral	N/A	Unknown	Unknown	Unknown	Unknown	Unknown	Unknown

Supplementary Table 1

ISPOCA- Instituto Superior Politecnico Do Cazenga	Sao Tome and Principe		http://www.ispoca.com/61.html	No	No	No	No	No	No
National Lyceum-Lyceum Correspondence	Sao Tome and Principe		http://www.lyceum.co.za/	No	No	No	No	No	No
Université Cheikh Anta Diop	Senegal	Dakar	https://www.ucad.sn/	Yes	Yes	Yes	Yes	No	No
Gaston Berger University	Senegal	Saint-Louis	http://www.ugb.sn/	Yes	Yes	No	Yes	No	No
Université Alioune DIOP de Bambey	Senegal	Diourbel	http://www.uadb.edu.sn/	Yes	Yes	No	Yes	No	No
Université de Thiès	Senegal	Thiès	https://www.univ-thies.sn/	Yes	No	No	No	No	No
Université du Sahel	Senegal	Dakar	http://unis.sn/	Yes	Yes	No	Yes	Yes	Yes
Université Dakar Bourguiba	Senegal	Dakar	http://udb-sn.com/	No	No	No	No	No	No
University of Seychilles	Senegal	Seychilles	http://www.unisey.ac.sc/	No	No	No	No	No	No
University of Seychelles	Seychelles	Anse Royale	http://www.unisey.ac.sc/	No	No	No	No	No	No
Njala University	Sierra Leone	Freetown, Western Area	http://njala.edu.sl/	Yes	Yes	Yes	Yes	No	No
University of Makeni	Sierra Leone	Northern Province	http://unimak.edu.sl/	No	No	Yes	No	No	No
Jaamacada Muqdisho	Somalia	Banaadir	http://mu.edu.so/	No	Yes	No	No	No	No
Jaamacada Kismaayo	Somalia	Lower Juba	http://www.kismayouniversity.com/	No	Yes	Yes	No	No	No
Jaamacada Camuud	Somalia	Awdal	http://amouduniversity.org//	No	Yes	Yes	No	No	No
Jaamacada Puntland State University	Somalia	Puntland	http://puntlandstateuniversity.com/	No	No	Yes	No	No	No
Jaamacada Burco	Somalia	Burao	http://universityofburao.com/	No	Yes	No	No	No	No

Supplementary Table 1

Jaamacada SIMAD	Somalia	Banaadir	http://simad.edu.so/	No	Yes	No	No	No	No
Jaamacada Bariga Afrika	Somalia	Bari	http://eastafricauniversity.net/	No	Yes	Yes	No	No	No
Jaamacada Soomaaliya	Somalia	Banaadir	http://www.uniso.edu.so/	No	Yes	Yes	No	No	No
Jaamacada Nugaal	Somalia	Sool	http://www.nugaaluniversity.com/	No	No	No	No	No	No
Jaamacada Golis	Somalia	Somaliland	http://www.gollisuniversity.com/	No	Yes	Yes	No	No	No
Jaamacada Unmada Soomaaliyeed	Somalia	Banaadir	http://snu.edu.so/	Yes	Yes	Yes	Yes	No	No
Jaamacada Hargeysa	Somalia	Somaliland	http://uoh-edu.net/	Yes	Yes	Yes	Yes	No	No
Jaamacada Badweynta Hindiya	Somalia	Banaadir	http://www.iou.edu.so/	No	Yes	Yes	No	No	No
Jaamacadda Teknoolajiyadda Somaliland	Somalia	Somaliland	http://www.somalilanduniversity.org/	Yes	No	No	Yes	No	No
Jaamacada Koonfur Galbeed Soomaaliya	Somalia	Baidoa Bay	http://uss-baidoa.org/	No	No	Yes	No	No	No
Jaamacada Plasma	Somalia	Banaadir	http://plasmauniversity.net/	No	Yes	Yes	No	No	No
Jaamacada Jamhuriya	Somalia	Banaadir	http://www.jamhuriyauniversity.com/	No	Yes	Yes	No	No	No
Jaamacada Jazeera	Somalia	Banaadir	http://jazeerauniversity.com/	No	Yes	Yes	No	No	No
University of Cape Town	South Africa	Cape Town	https://www.uct.ac.za/	Yes	Yes	Yes	Yes	Yes	Yes
University of South Africa	South Africa	Pretoria	http://www.unisa.ac.za/	Yes	Yes	Yes	Yes	Yes	Yes
University of Pretoria	South Africa	Gauteng	http://www.universityofpretoria.co.za/	Yes	Yes	Yes	Yes	Yes	Yes

Supplementary Table 1

Universiteit Stellenbosch	South Africa	Western Cape	http://www.sun.ac.za/	Yes	Yes	Yes	Yes	Yes	Yes
University of the Witwatersrand	South Africa	Gauteng	http://www.wits.ac.za/	Yes	Yes	Yes	Yes	Yes	Yes
North-West University	South Africa	North West	http://www.nwu.ac.za/	Yes	No	No	Yes	No	No
University of KwaZulu-Natal	South Africa	KwaZulu-Natal	http://chs.ukzn.ac.za/	Yes	No	Yes	Yes	Yes	Yes
Rhodes University	South Africa	Eastern Cape	https://www.ru.ac.za/	Yes	No	No	Yes	Yes	Yes
University of Johannesburg	South Africa	Johannesburg	https://www.uj.ac.za/	Yes	No	Yes	Yes	Yes	Yes
University of the Western Cape	South Africa	Bellville	https://www.uwc.ac.za	Yes	No	Yes	Yes	Yes	Yes
Nelson Mandela Metropolitan University	South Africa	Eastern Cape	https://www.nmmu.ac.za/	Yes	Yes	No	Yes	Yes	Yes
Cape Peninsula University of Technology	South Africa	Western Cape	http://www.cput.ac.za/	Yes	No	No	Yes	No	No
Universiteit van die Vrystaat	South Africa	Free State	http://www.ufs.ac.za/	No	Yes	Yes	Yes	Yes	Yes
Durban University of Technology	South Africa	Durban	http://www.dut.ac.za/	Yes	Yes	Yes	Yes	Yes	Yes
Tshwane University of Technology	South Africa	Gauteng	http://www.tut.ac.za/	Yes	Yes	Yes	Yes	Yes	Yes
University of Fort Hare	South Africa	Eastern Cape	http://www.ufh.ac.za/	Yes	Yes	Yes	Yes	Yes	Yes
Mangosuthu University of Technology	South Africa	KwaZulu-Natal	http://www.ufh.ac.za/	No	Yes	No	No	No	No
University of Zululand	South Africa	KwaZulu-Natal	http://www.unizulu.ac.za/	Yes	No	No	Yes	Yes	Yes
Central University of Technology	South Africa	Free State	http://www.cut.ac.za/	No	No	No	No	No	No

Supplementary Table 1

Vaal University of Technology	South Africa	Gauteng	http://www.vut.ac.za/	Yes	No	No	Yes	No	No
University of Venda	South Africa	Limpopo	http://www.univen.ac.za /	Yes	No	Yes	Yes	Yes	No
University of Limpopo	South Africa	Limpopo	https://www.ul.ac.za/	No	Yes	Yes	No	No	No
Walter Sisulu University	South Africa	Mtatha	http://www.wsu.ac.za/	No	Yes	Yes	Yes	Yes	No
University of Juba	South Sudan	Juba	http://jubauni.net/	Yes	Yes	No	Yes	No	No
John Garang Memorial	South Sudan	Jonglei	http://www.jgmust-	Yes	No	No	Yes	No	No
University of Khartoum	Sudan	Khartoum	http://www.uofk.edu/	No	Yes	Yes	No	No	No
Sudan University of	Sudan	Khartoum	http://www.sustech.edu/	No	Yes	No	No	No	No
International University	Sudan	Khartoum	http://en.iua.edu.sd/	No	Yes	No	No	No	No
University of Science	Sudan	Omdurman	http://www.sustech.edu/	No	Yes	No	No	No	No
Ahamad University For	Sudan	Khartoum	http://ahfad.edu.sd/	No	Yes	Yes	No	No	No
University of Gezira	Sudan	Al Jazirah	http://uofg.edu.sd/en/	Yes	Yes	Yes	Yes	Yes	Yes
University of Shendi	Sudan	River Nile	http://www.ush.sd/ar/	No	Yes	Yes	No	No	No
Neelain University	Sudan	Khartoum	http://neelain.edu.sd/	Yes	Yes	Yes	Yes	Yes	Yes
Omdurman Islamic	Sudan	Khartoum	http://www.oiu.edu.sd/e	No	Yes	No	No	No	No
University of Dongola	Sudan	Dongola	http://uofd.edu.sd/	No	Yes	No	No	No	No
National Ribat	Sudan	Khartoum	http://www.ribat.edu.sd/	No	Yes	No	No	No	No
Nile Valley University	Sudan	Khartoum	http://www.nilevalley.ed	No	Yes	No	No	No	No
Red Sea University	Sudan	Port Sudan	http://rsu.edu.sd/en/	Yes	Yes	No	Yes	No	No
Karary University	Sudan	Omdurman	http://www.karary.edu.s	No	Yes	No	No	No	No
Al Zaiem Al Azhari	Sudan	Khartoum	http://aau.edu.sd/	No	Yes	Yes	Yes	No	No
University of Nyala	Sudan	Nyala	http://www.nyalau.edu.s	No	Yes	No	No	No	No
University of the Holy	Sudan	Omdurman	http://quran-unv.edu.sd/	No	No	No	No	No	No
University of Medical	Sudan	Khartoum	http://www.umst-	No	Yes	No	No	No	No
Future University	Sudan	Khartoum	http://www.futureu.edu.	No	No	No	No	No	No
University of Gadarif	Sudan	Gadaref	http://www.gaduniv.edu.	No	No	No	No	No	No

Supplementary Table 1

University of Kordofan	Sudan	El-Obeid	http://kordofan.edu.sd/	No	Yes	No	No	No	No
Kassala University	Sudan	Kassala	http://kassalauni.edu.sd/	No	Yes	No	No	No	No
University of Bakht	Sudan	El-Dueim	http://www.uofb.edu.sd/	Yes	Yes	No	No	No	No
Sudan Academy of	Sudan	Khartoum	http://www.sas.edu.sd/	Yes	Yes	No	Yes	Yes	Yes
Al Fashir University	Sudan	Khartoum	http://www.fashir.edu.sd	No	Yes	No	No	No	No
University of Zalingei	Sudan	Khartoum	http://zalingei.edu.sd/	No	Yes	No	No	No	No
Omdurman Ahlia	Sudan	Omdurman	http://oau.edu.sd/site/in	No	Yes	No	No	No	No
University of Blue Nile	Sudan	Ad Damazin	http://uobn.edu.sd/index	No	No	No	No	No	No
National University-Sudan	Sudan	Khartoum	http://www.nu.edu.sd/	No	Yes	Yes	No	No	No
West Kordufan University	Sudan	South Kordufan	http://www.wku.edu.sd/	No	Yes	No	No	No	No
University of Swaziland	Swaziland	Kwaluseni	http://www.uniswa.sz/	No	No	No	No	No	No
Mzumbe University	Tanzania	Morogoro	http://site.mzumbe.ac.tz/	No	Yes	Yes	No	No	No
Sokoine University of Agriculture	Tanzania	Morogoro	http://www.suanet.ac.tz/index.php/education/programmes-offered-at-sua	Yes	Yes	Yes	Yes	Yes	Yes
Muhimbili University of Health and Allied Science	Tanzania	Ilala	http://www.muhas.ac.tz/	Yes	Yes	Yes	Yes	Yes	No
St. Augustine University of Tanzania	Tanzania	Dodoma	https://saut.ac.tz/	No	No	No	No	No	No
Ardhi University	Tanzania	Dar Es Salaam	http://www.aru.ac.tz/	No	No	No	No	No	No
Mbeya University of Science and Technology	Tanzania	Myeba	http://www.mustnet.ac.tz/	No	No	No	No	No	No
Tumaini University of Makumira	Tanzania	Iringa	http://www.makumira.ac.tz/	No	No	No	No	No	No
St.John's University of Tanzania	Tanzania	Dodoma	http://www.sjut.ac.tz/	No	No	Yes	No	No	No

Supplementary Table 1

State University of Zanzibar	Tanzania	Dar Es Salaam	http://www.suza.ac.tz/	No	Yes	Yes	No	No	No
Mkwawa University College of Education	Tanzania	Iringa	http://www.muce.ac.tz/	No	No	No	No	No	No
Dar Es Salaam University College of Education	Tanzania	Dar Es Salaam	http://duce.ac.tz/	Yes	No	No	Yes	No	No
Catholic University of Health And Allied Science	Tanzania	Mwanza	http://www.bugando.ac.tz/	No	Yes	Yes	No	No	No
Moshi Co-operative University	Tanzania	Moshi	http://www.mocu.ac.tz/	No	No	No	No	No	No
Zanzibar University	Tanzania	Zanzibar	http://www.zanvarsity.ac.tz/	No	Yes	No	No	No	No
University of Arusha	Tanzania	Arusha	http://www.uoa.ac.tz/	No	No	No	No	No	No
Sebastian Kolowa Memorial University (SEKOMU)	Tanzania	Lushoto	http://www.sekomu.ac.tz/	Yes	Yes	No	Yes	No	No
Mount Meru University	Tanzania	Arusha	http://www.mmu.ac.tz/	No	No	No	No	No	No
Hubert Kairuki Memorial University	Tanzania	Dar Es Salaam	http://www.hkmu.ac.tz/	No	Yes	Yes	No	No	No
Stella Maris Mtwara University College	Tanzania	Mtwara	http://www.stemmuco.ac.tz/	No	No	No	No	No	No
Jordan University College	Tanzania	Morogoro	http://www.juco.ac.tz/	No	No	No	No	No	No
Muslim University of Morogoro	Tanzania	Morogoro	http://www.mum.ac.tz/	-	-	-	-	-	-
Nelson Mandela African Institute of Science and Technology	Tanzania	Arusha	http://www.nm-aist.ac.tz/	No	No	No	No	No	No

Supplementary Table 1

Kampala International University Dar es Salaam College	Tanzania	Dar Es Salaam	http://www.kiu.ac.tz/	No	Yes	No	No	No	No
Eastern and Southern African Management Institute	Tanzania	Arusha	http://www.esami-africa.org/	No	No	No	No	No	No
International Medical and Technological University	Tanzania	Dar Es Salaam	www.imtu.ed	No	Yes	No	No	No	No
Stefano Moshi Memorial	Tanzania	Moshi	http://www.smmuco.ac.tz/	No	No	No	No	No	No
St. Francis University College of Health and Allied Sciences	Tanzania	Ifakara	http://www.sfuchas.ac.tz/	No	Yes	Yes	No	No	No
Josiah Kibira University College	Tanzania	Bukoba	http://www.jokuco.ac.tz/	No	No	No	No	No	No
Archbishop Mihayo University College of Tabora	Tanzania	Tabora	http://www.amucta.ac.tz/	No	No	No	No	No	No
Kisongo Academic College	Tanzania	Arusha	http://www.kisongocollege.com/	No	No	No	No	No	No
Eckernforde Tanga University	Tanzania	Tanga	http://www.etu.ac.tz/	No	Yes	No	No	No	No
United African University of Tanzania	Tanzania	Dar Es Salaam	http://www.uaut.ac.tz/	No	No	No	No	No	No
University of Bagamoyo	Tanzania	Dar Es Salaam	http://www.uob.ac.tz/	Yes	No	No	Yes	Yes	Yes
Teofilo Kisanji University	Tanzania	Mbeya	http://www.teku.ac.tz/	No	No	No	No	No	No
St. Joseph University in Tanzania	Tanzania	Dar Es Salaam	http://www.sjuit.ac.tz/	No	Yes	No	No	No	No

Supplementary Table 1

Université de Lomé	Togo	Lomé	http://www.univ-lome.tg/	Yes	Yes	No	Yes	Yes	Yes
Université de Kara	Togo	Kara	http://www.unikara.org/	No	Yes	Yes	No	No	No
Makerere University	Uganda	Central Region	https://www.mak.ac.ug/	Yes	Yes	No	Yes	No	No
Ndejje University	Uganda	Central Region	http://www.ndejjeuniversity.ac.ug/	Yes	No	No	Yes	No	No
Uganda Christian University	Uganda	Central Region	http://ucu.ac.ug/	Yes	Yes	Yes	Yes	Yes	Yes
Kampala International University	Uganda	Central Region	http://kiu.ac.ug/	No	Yes	Yes	No	No	No
Kyambogo University	Uganda	Central Region	http://www.kyu.ac.ug/	Yes	No	Yes	Yes	No	No
Uganda Technology and Management University	Uganda	Central Region	http://www.utamu.ac.ug/	No	No	No	No	No	No
Uganda Martyrs University	Uganda	Central Region	http://www.umu.ac.ug/	No	No	Yes	No	No	No
Victoria University	Uganda	Central Region	http://www.vu.ac.ug/	No	No	Yes	No	No	No
International University of East Africa	Uganda	Central Region	http://iuea.ac.ug/	No	No	No	No	No	No
Muteesa I Royal International	Uganda	Central Region	http://mru.ac.ug/home	No	No	No	No	No	No
Cavendish University Uganda	Uganda	Central Region	http://www.cavendish.ac.ug/	No	No	Yes	No	No	No
Kampala University	Uganda	Central Region	http://www.ku.ac.ug/	No	No	No	No	No	No
St. Augustine International University	Uganda	Central Region	http://www.saiu.ac.ug/	No	Yes	Yes	No	No	No
St. Lawrence University	Uganda	Central Region	http://www.slau.ac.ug/	No	No	No	No	No	No
Islamic University of Uganda	Uganda	Eastern Region	http://www.iuiu.ac.ug/	No	Yes	No	No	No	No
Busitema University	Uganda	Eastern Region	http://busitema.ac.ug/	No	Yes	Yes	No	No	No
Livingstone University	Uganda	Eastern Region	http://livingstone.ac.ug/	No	No	No	No	No	No

Supplementary Table 1

Busoga University	Uganda	Eastern Region	http://www.busogauniversity.ac.ug/	No	No	No	No	No	No
Kumi University	Uganda	Eastern Region	N/A	No	Yes	No	No	No	No
Soroti University	Uganda	Eastern Region	http://www.sun.ac.ug/	No	Yes	Yes	No	No	No
Guli University	Uganda	Northern Region	http://www.gu.ac.ug/	Yes	Yes	Yes	Yes	No	No
Muni University	Uganda	Northern Region	http://www.muni.ac.ug/	No	No	No	No	No	No
All Saints University	Uganda	Northern Region	http://www.asul.ac.ug/	No	No	No	No	No	No
Mbarara University of Science and Technology	Uganda	Western Region	http://www.must.ac.ug/	No	Yes	Yes	No	No	No
Mountains of the Moon University	Uganda	Western Region	http://mmu.ac.ug/	No	Yes	Yes	No	No	No
Kabale University	Uganda	Western Region	http://www.kab.ac.ug/	No	No	No	No	No	No
African Rural University	Uganda	Western Region	http://aru.ac.ug/	No	No	No	No	No	No
Bishop Stuart University	Uganda	Western Region	http://www.bsu.ac.ug/	No	No	Yes	No	No	No
Uganda Pentecostal University	Uganda	Western Region	http://upu.ac.ug/	No	No	No	No	No	No
The Copperbelt University	Zambia	Copperbelt	http://www.cbu.edu.zm/	Yes	Yes	No	Yes	Yes	No
Northrise University	Zambia	Copperbelt	https://northrise.org/university-campus/	No	No	No	No	No	No
Zambia Catholic University	Zambia	Copperbelt	http://www.zcuniversity.edu.zm/	No	No	No	No	No	No
Copperstone University	Zambia	Copperbelt	N/A	No	No	No	No	No	No
University of Zambia	Zambia	Lusaka	https://www.unza.zm/	Yes	Yes	Yes	Yes	No	No
University of Lusaka	Zambia	Lusaka	http://www.unilus.ac.zm/	No	No	No	No	No	No
Cavendish University Zambia	Zambia	Lusaka	http://www.cavendishza.org/	No	No	No	No	No	No
DMI- St. Eugene University	Zambia	Lusaka	http://www.dmisteugene.org/	No	No	No	No	No	No

Supplementary Table 1

Lusaka Apex Medical University	Zambia	Lusaka	http://www.lamu.edu.zm/	No	Yes	No	No	No	No
University of Zimbabwe	Zimbabwe	Harare	http://www.uz.ac.zw/	Yes	Yes	Yes	Yes	No	No
Women's University in Africa	Zimbabwe	Harare	http://www.wua.ac.zw/	No	No	No	No	No	No
Harare Institute of Technology	Zimbabwe	Harare	http://www.hit.ac.zw/	No	No	No	No	No	No
Catholic University in	Zimbabwe	Harare	http://www.cuz.ac.zw/	No	No	No	No	No	No
National University of Science and Technology	Zimbabwe	Matabeleland North	http://www.nust.ac.zw/	No	Yes	No	No	No	No
Solusi University	Zimbabwe	Matabeleland North	http://solusi.ac.zw/	Yes	No	No	Yes	No	No
Lupane State University	Zimbabwe	Matabeleland North	https://lsu.ac.zw/	No	No	No	No	No	No

Supplementary Table 1

Medicine	Public health, Undergraduate Level	Public Health, Masters Level	Public health, doctoral level	Vaccinology	Microbiology/ Bacteriology	Virology	Immunology	Epidemiology	Health economics	Health policy
No	Yes	Yes	No	No	No	No	No	Yes	Yes	Yes
Yes	Yes	Yes	Yes	Don't know	Yes	No	No	Yes	No	No
No	No	No	No	No	No	No	No	No	No	No
No	No	No	No	Don't know	No	No	No	No	No	No
No	Yes	No	No	No	No	Yes	No	No	No	No
Yes	No	No	No	Don't know	No	Yes	No	No	No	No
Yes	No	No	No	No		Yes	Yes	Yes	No	No
No	No	No	No	No	No	No	No	No	No	No
Yes	No	No	No	Don't know	No	No	No	No	No	No
Yes	No	No	No	No	No	No	No	No	No	No
Yes	No	No	No	No	Yes	No	Yes	Yes	No	No
No	No	No	No	No	No	No	No	No	No	No
Yes	No	No	No	No	Yes	No	No	No	No	No
Yes	No	No	No	No	Yes	Yes	Yes	Yes	No	No
No	No	No	No		No	No	No	No	No	No
No	No	No	No	No	No	No	No	No	No	No
Yes	Yes	No	No	No	Yes	No	No	Yes	No	No

Supplementary Table 1

No	No	No	No	No	No	No	No	No	No	No
No	No	No	No	No	No	No	No	No	No	No
No	No	No	No	No	No	No	No	No	No	No
No	No	No	No	Don't know	No	No	No	No	No	No
No	No	No	No	No	No	No	No	No	No	No
No	No	No	No	No	No	No	No	No	No	No
Yes	Yes	No	No	Yes	Yes	Yes	Yes	No	Yes	No
No	No	No	No	No	Yes	Yes	Yes	Yes	No	No
No	Yes	No	No	No	No	No	No	Yes	No	No
No	No	No	No	No	No	No	No	No	No	No
No	No	No	No	No	Yes	No	Yes	No	No	No
Yes	No	No	No	No	Yes	No	No	No	No	No

Supplementary Table 1

Yes	No	No	No	Yes	No	No	Yes	No	No	No
No	No	No	No	No	No	No	No	No	No	No
Yes	No	No	No	Don't know	No		No	No	No	No
No	No	No	No	No	No	No	No	No	No	No
No	No	No	No	No	No	No	No	No	No	No
No	No	No	No	No	No	No	No	No	No	No
No	No	No	No	No	No	No	No	No	No	No
No	No	No	No	No	No	No	No	No	No	No
No	No	No	No	No	No	No	No	No	No	No
No	No	No	No	No	No	No	No	No	No	No
Yes	No	No	No	No	Yes	No	No	No	No	No
Yes	Yes	Yes	Yes	No	Yes	No	Yes	Yes	No	No
No	No	No	No	No	No	No	No	No	No	No
No	No	No	No	No	No	No	No	No	No	No
Yes	No	No	No	No	No	No	No	No	No	No
Yes	Yes	No	No	No	No	No	No	No	No	No

Supplementary Table 1

Yes	No	No	No	No	Yes	Yes	Yes	Yes	No	No
No	No	No	No	No	No	Yes	No	No	No	No
Yes	No	Yes	No	No	Yes	No	Yes	Yes	Yes	No
Yes	No	No	No	No	No	No	Yes	No	No	No
Yes	Yes	Yes	Yes	No	Yes	No	No	Yes	No	No
Yes	No	Yes	No	No	Yes	No	Yes	Yes	Yes	No
Yes	No	No	No	No	No	Yes	No	No	No	No
Yes	No	No	No	No	Yes	Yes	Yes	No	No	No
No	No	No	No	No	Yes	No	Yes	Yes	No	No
No	No	No	No	No	No	No	No	No	No	No
Yes	Yes	No	No	No	Yes	Yes	No	Yes	Yes	No
Yes	Yes	No	No	No	Yes	Yes	Yes	Yes	Yes	Yes
No	No	No	No	No	Yes	No	No	No	No	No
Yes	Yes	No	No	No	Yes	Yes	No	Yes	Yes	No
No	No	No	No	No	No	Yes	No	No	No	No
No	No	No	No	No	No	No	No	No	No	No

Supplementary Table 1

No	Yes	Yes	No	No	No	Yes	No	No	No	Yes
Yes	Yes	No	No	No	Yes	No	No	Yes	No	No
No	No	No	No	No	No	No	No	No	No	No
No	No	No	No	No	No	No	No	No	No	No
No	No	No	No	No	No	No	No	No	No	No
No	No	No	No	No	No	No	No	No	No	No
No	No	No	No	No	No	No	No	No	No	No
No	No	No	No	No	No	No	No	No	No	No
Yes	Yes	No	No	No	Yes	No	No	No	No	No
Yes	Yes	No	No	No	Yes	Yes	Yes	Yes	Yes	Yes
Yes	Yes	No	No	No	No	No	No	No	No	No
Unknown	Unknown	Unknown	Unknown	Unknown	Unknown	Unknown	Unknown	Unknown	Unknown	Unknown
Yes	Yes	No	No	No	Yes	Yes	Yes	Yes	No	No
Yes	No	No	No	No	No	No	No	No	No	No
Yes	Yes	No	No	No	Yes	No	Yes	Yes	Yes	No

Supplementary Table 1

Yes	No	No	No	No	Yes	No	Yes	Yes	No	No
No	No	No	No	No	No	No	No	No	No	No
Yes	Yes	No	No	No	No	No	Yes	No	No	No
Yes	No	No	No	No	Yes	No	No	Yes	No	No
No	No	No	No	No	No	No	No	No	No	No
Yes	No	No	No	No	Yes	Yes	Yes	Yes	No	No
Yes	Yes	No	No	No	Yes	Yes	No	No	No	No
No	No	No	No	No	Yes	Yes	No	No	No	No
Yes	No	No	No	No	No	Yes	Yes	No	No	No
Yes	Yes	Yes	Yes	No	No	Yes	Yes	Yes	No	No
Yes	No	No	No	No	No	No	No	No	No	No
Yes	No	No	No	No	No	Yes	No	No	No	No
Yes	No	No	No	No	No	No	No	No	Yes	No
Yes	Yes	No	No	No	No	Yes	No	No	No	No
No	No	No	No	No	No	No	No	No	No	No
Yes	Yes	No	No	No	Yes	Yes	Yes	Yes	No	No

Supplementary Table 1

Yes	No	No	No	No	No	No	No	No	No	No
Yes	No	No	No	No	No	No	No	No	No	No
Yes	Yes	No	No	No	No	No	No	Yes	No	No
Yes	Yes	Yes	No	No	No	No	No	No	No	No
Yes	Yes	No	No	No	No	No	Yes	Yes	No	No
No	No	No	No	No	No	No	No	No	No	No
No	No	No	No	No	No	No	No	No	No	No
No	No	No	No	No	No	No	No	No	No	No
No	No	No	No	No	Yes	Yes	Yes	Yes	Yes	Yes
Yes	No	No	No	No	No	No	No	No	No	No
No	No	No	No	No	No	No	No	No	No	No
No	No	No	No	No	No	No	No	No	No	No
Yes	Yes	No	No	No	Yes	No	Yes	Yes	No	No
Yes	Yes	No	No	No	Yes	Yes	Yes	Yes	Yes	No
Yes	Yes	No	No	No	Yes	Yes	Yes	Yes	No	No
Yes	Yes	Yes	No.	No	Yes	No	Yes	Yes	No.	No
Yes	Yes	No	No.	No	Yes	Yes	Yes	Yes	Yes	Yes

Supplementary Table 1

No	No	No	No	No	No	No	No	No	No	No
Yes	No	No	No	No	Yes	No	Yes	Yes	No	No
Yes	Yes	No	No	No	Yes	No	No	Yes	No	No
Yes	Yes	Yes	Yes	No	Yes	Yes	Yes	Yes	No	No
Yes	Yes	No	No	No	Yes	Yes	Yes	Yes	No	No
Yes	Yes	No	No	No	Yes	Yes	Yes	Yes	No	No
Yes	Yes	Yes	Yes	No	Yes	Yes	Yes	Yes	No	No
No	No	No	No	No	Yes	No	No	No	No	No
Yes	Yes	No	No	No	Yes	Yes	Yes	Yes	No	No
Yes	Yes	No	No	No	Yes	No	Yes	No	No	No
Yes	Yes	Yes	No	Yes	Yes	Yes	Yes	Yes	No	No
Yes	Yes	No	No	No	Yes	Yes	Yes	Yes	Yes	No
No	No	No	No	No	No	No	No	No	No	No
Yes	Yes	No	No	No	Yes	No	Yes	Yes	No	No
No	No	No	No	No	No	No	No	No	No	No
Yes	Yes	Yes	No	No	No	No	Yes	No	No	No
No	No	No	No	No	Yes	Yes	Yes	No	No	No
No	Yes	No	No	No	No	Yes	No	Yes	No	No

Supplementary Table 1

Yes	Yes	No	No	No	Yes	No	Yes	Yes	No	No
No	Yes	No	No	No	Yes	No	Yes	No	No	No
No	Yes	No	No	No	Yes	No	Yes	Yes	No	No
No	No	No	No	No	No	No	No	No	No	No
Yes	No	No	No	No	Yes	Yes	Yes	No	No	No
Yes	Yes	No	N	No	Yes	No	No	No	No	No
No	No	No	No	No	No	No	No	No	No	No
No	No	No	No	No	No	No	No	No	No	No
No	No	No	No	No	No	No	No	No	No	No
No	No	No	No	No	No	No	No	No	No	No
No	Yes	Yes	No	No	No	No	No	Yes	No	No
Yes	Yes	No	No	No	No	No	No	No	No	No
Yes	No	No	No	No	Yes	No	No	No	No	No
Yes	Yes	Yes	Yes	No	Yes	Yes	Yes	Yes	Yes	Yes

Supplementary Table 1

Yes	Yes	Yes	Yes	No	Yes	Yes	Yes	Yes	Yes	Yes
No	No	No	No	No	No	No	No	No	No	No
No	No	No	No	No	No	No	Yes	Yes	No	No
Yes	No	No	No	No	No	No	No	Yes	No	No
No	No	No	No	No	No	No	No	No	No	No
No	No	No	No	No	No	No	No	No	No	No
No	No	No	No	No		No	Yes	No	No	No
No	No	No	No	No	No	No	No	No	No	No
Yes	No	No	No	No	Yes	No	No	No	No	No
No	No	No	No	No	No	No	No	No	No	No
No	No	No	No	No	No	No	No	No	No	No
Yes	Yes	No	No	No	No	No	No	No	No	No
No	No	No	No	No	No	No	No	No	No	No
No	No	No	No	No	No	No	No	No	No	No
No	No	No	No	No	No	No	No	No	No	No
No	No	No	No	No	No	No	No	No	No	No

Supplementary Table 1

No	No	No	No	No	No	No	No	No	No	No
No	No	No	No	No	No	No	No	No	No	No
Yes	No	No	No	No	Yes	Yes	Yes	Yes	No	No
Yes	Yes	No	No	No	Yes	Yes	Yes	Yes	No	No
No	No	No	No	No	No	No	No	No	No	No
No	No	No	No	No	No	No	No	No	No	No
No	No	No	No	No	No	No	No	No	No	No
No	No	No	No	No	No	No	No	No	No	No
Yes	No	No	No	No	Yes	No	No	No	No	No
Yes	Yes	Yes	Yes	Yes	Yes	Yes	Yes	Yes	Yes	No
No	No	No	No	No	No	No	No	No	No	No
No	No	No	No	No	No	No	No	No	No	No
No	No	No	No	No	No	No	No	No	No	No
No	No	No	No	No	No	No	No	No	No	No
No	No	No	No	No	No	No	No	No	No	No

Supplementary Table 1

Yes	No	No	No	No	Yes	No	Yes	No	No	No
No	Yes	No	No	No	No	Yes	Yes	No	Yes	Yes
Yes	No	No	No	No	No	No	Yes	No	No	No
No	No	No	No	No	No	No	No	No	No	No
No	No	No	No	No	No	No	No	No	No	No
No	No	No	No	No	No	No	No	No	No	No
Yes	No	No	No	No	No	Yes	Yes	No	No	No
No	Yes	No	No	No	No	No	No	No	No	No
No	No	No	No	No	No	No	No	No	No	No
Yes	No	No	No	No	Yes	No	Yes	No	No	No
No	No	No	No	No	No	No	No	No	No	No
No	No	No	No	No	No	No	No	No	No	No
No	No	No	No	No	No	No	No	No	No	No
No	Yes	No	No	No	Yes	Yes	Yes	Yes	No	Yes
No	No	No	No	No	No	No	No	No	No	No
No	No	No	No	No	No	No	No	No	No	No

Supplementary Table 1

No	No	No	No	No	No	No	No	No	No	No
No	No	No	No	No	No	No	No	No	No	No
No	No	No	No	No	No	No	No	No	No	No
Yes	No	No	No	No	No	Yes	No	No	No	No
No	No	No	No	No	No	No	No	No	No	No
Yes	No	No	No	No	Yes	Yes	Yes	No	No	No
No	No	No	No	No	No	No	No	No	No	No
No	No	No	No	No	No	No	No	No	No	No
No	No	No	No	No	No	No	No	No	No	No
No	No	No	No	No	No	No	No	No	No	No
No	Yes	No	No	No	Yes	No	No	No	No	No
No	No	No	No	No	No	No	No	No	No	No
No	No	No	No	No	No	No	No	No	No	No
No	No	No	No	No	No	No	No	No	No	No
No	No	No	No	No	No	No	No	No	No	No

Supplementary Table 1

Yes	No	No	No	No	Yes	No	No	No	No	No
Yes	No	No	No	No	No	No	Yes	No	No	No
Unknown	Unknown	Unknown	Unknown	Unknown	Unknown	Unknow n	Unknown	Unknown	Unknown	Unknown
Basically, Nursing	No	No	No	No	Yes	Yes	No	No	No	No
Yes	No	No	No	No	No	No	No	No	No	No
No	No	No	No	No	No	No	No	No	No	No
No	No	No	No	No	No	No	No	No	No	No
Yes	No	No	No	No	No	No	Yes	Yes	No	No
No	No	No	No	No	Yes	Yes	No	No	No	No
No	No	No	No	No	No	No	No	No	No	No
Yes	No	No	No	No	No	No	No	No	No	No

Supplementary Table 1

No	No	No	No	No	No	No	No	No	No	No
No	No	No	No	No	No	No	No	No	No	No
No	No	No	No	No	No	No	No	No	No	No
Yes	No	No	No	No	Yes	No	No	No	No	No
No	No	No	No	No	No	No	No	No	No	No
No	No	No	No	No	No	No	No	No	No	No
Yes	Yes	Yes	Yes	Yes	Yes	Yes	Yes	Yes	Yes	Yes
Yes	No	Yes	No	Yes	Yes	Yes	Yes	Yes	No	No
Yes	Yes	Yes	Yes	Yes	Yes	Yes	Yes	Yes	Yes	Yes
Yes	Yes	Yes	Yes	Yes	Yes	Yes	Yes	Yes	No	No
No	No	No	No	No	No	No	No	No	No	No
Yes	Yes	Yes	Yes	Yes	Yes	Yes	Yes	Yes	No	No
No	No	No	No	No	No	No	No	No	No	No
Yes	Yes	Yes	Yes	No	Yes	Yes	Yes	Yes	No	No

Supplementary Table 1

No	No	No	No	No	No	No	No	No	No	No
Yes	Yes	Yes	Yes	Yes	No	Yes	Yes	Yes	No	No
Yes	Yes	Yes	Yes	No	Yes	Yes	Yes	Yes	Yes	Yes
Yes	Yes	Yes	No	No	Yes	No	Yes	No	No	No
No	No	No	No	No	No	No	Yes	No	No	No
Yes	Yes	Yes	Yes	Yes	Yes	Yes	Yes	Yes	No	No
No	No	No	No	No	No	No	No	No	No	No
No	No	No	No	No	No	No	No	No	No	No
No	Yes	No	No	No	No	Yes	Yes	Yes	Yes	No
No	No	No	No	No	No	No	No	No	No	No
Yes	No	No	No	No	No	Yes	Yes	Yes	No	No
Yes	Yes	No	No	No	Yes	Yes	Yes	Yes	No	No
Yes	Yes	Yes	No	No	Yes	Yes	Yes	Yes	No	No
Yes	No	No	No	No	Yes	Yes	Yes	Yes	No	No
No	No	No	No	No	No	No	No	No	No	No
No	Yes	Yes	Yes	No	Yes	Yes	Yes	Yes	No	No
No	No	No	No	No	No	No	No	No	No	No

Supplementary Table 1

No	No	No	No	No	Yes	Yes	Yes	Yes	No	No
No	No	No	No	No	No	No	No	No	No	No
No	Yes	Yes	Yes	No	Yes	No	Yes	Yes	No	No
Yes	Yes	Yes	Yes	No	Yes	No	Yes	Yes	Yes	No
No	Yes	Yes	Yes	No	Yes	No	Yes	Yes	No	No
Yes	Yes	Yes	Yes	No	Yes	No	Yes	No	Yes	No
No	No	No	No	No	No	No	Yes	Yes	Yes	No
Yes	Yes	Yes	Yes	No	Yes	Yes	Yes	Yes	No	No
No	No	No	No	No	Yes	No	Yes	Yes	No	No
No	No	No	No	No	Yes	Yes	Yes	Yes	Yes	Yes
Yes	Yes	Yes	Yes	No	Yes	Yes	Yes	Yes	Yes	Yes
No	Yes	No	No	No	No	No	No	No	No	No
No	No	No	No	No	No	No	No	No	No	No
No	No	No	No	No	No	No	No	No	No	No
No	No	No	No	No	No	No	No	No	No	No

Supplementary Table 1

No	No	No	No	No	No	No	No	No	No	No
No	No	No	No	No	No	No	No	No	No	No
No	No	No	No	No	No	No	No	No	No	No
No	No	No	No	No	No	No	No	No	No	No
No	Yes	Yes	Yes	Yes	Yes	No	Yes	Yes	Yes	Yes
Yes	No	No	No	No	No	Yes	Yes	Yes	No	No
Yes	No	No	No	No	No	No	Yes	Yes	No	No
No	No	No	No	No	No	No	No	No	No	No
No	Yes	No	No	No	No	No	Yes	Yes	No	No
No	No	No	No	No	No	No	No	No	No	No
No	No	No	No	No	No	No	No	No	No	No
Yes	No	No	No	No	No	No	Yes	Yes	No	No
No	No	No	No	No	No	No	No	No	No	No
Yes	Yes	Yes	No	No	No	No	No	Yes	No	No
No	No	No	No	No	No	No	No	No	No	No
Yes	No	No	No	No	No	Yes	Yes	Yes	No	No
Yes	Yes	Yes	Yes	No	Yes	No	Yes	Yes	No	No

Supplementary Table 1

Yes	No	No	No	No	Yes	No	No	Yes	No	No
Yes	No	No	Yes	No	Yes	No	No	Yes	No	No
No	No	No	No	No	No	No	No	No	No	No
No	No	No	No	No	No	No	No	No	No	No
Yes	No	Yes	No	No	Yes	No	Yes	Yes	No	Yes
Yes	No	No	No	No	Yes	No	No	No	No	No
No	No	No	No	No	Yes	No	Yes	Yes	No	No
No	No	No	No	No	Yes	No	Yes	No	No	No
No	No	No	No	No	No	No	No	No	No	No
No	No	No	No	No	No	No	No	No	No	No
No	No	No	No	No	No	No	No	No	No	No
No	No	No	No	No	No	No	No	No	No	No
No	No	No	No	No	Yes	Yes	Yes	No	No	No
Yes	Yes	No	No	No	No	Yes	Yes	Yes	No	No

Supplementary Table 1

No	No	No	No	No	No	No	No	No	No	No
No	No	No	No	No	No	No	No	No	No	No
No	Yes	Yes	No	No	Yes	No	No	Yes	Yes	Yes
No	No	No	No	No	Yes	No	No	No	No	No
No	No	No	No	No	No	No	No	No	No	No
No	No	No	No	No	No	No	No	No	No	No
Yes	No	No	No	No	Yes	No	Yes	Yes	No	No
No	No	No	No	No	No	No	No	No	No	No
No	No	No	No	No	No	No	No	No	No	No
No	No	No	No	No	No	No	No	No	No	No
Yes	Yes	No	No	No	Yes	No	No	No	No	No
No	No	No	No	No	No	No	No	No	No	No
No	No	No	No	No	No	No	No	No	No	No
Yes	No	Yes	No	Yes	Yes	Yes	Yes	Yes	Yes	Yes
No	No	No	No	No	No	No	No	No	No	No

Supplementary Table 1

Yes	Yes	Yes	Yes	No	No	Yes	Yes	No	Yes	No
No	No	No	No	No	No	No	No	No	No	No
Yes	Yes	No	No	No	Yes	Yes	No	Yes	Yes	No
Yes	No	No	No	No	Yes	Yes	Yes	Yes	No	No
Yes	No	Yes	No	No	No	No	No	No	No	No
Yes	Yes	Yes	Yes	No	Yes	Yes	Yes	Yes	No	Yes
Yes	No	No	No	No	No	No	Yes	Yes	No	No
Yes	No	No	No	No	Yes	No	No	No	No	No
No	Yes	Yes	No	No	No	No	No	No	No	No
Yes	Yes	Yes	Yes	Yes	Yes	Yes	Yes	Yes	Yes	Yes
Yes	Yes	Yes	Yes	Yes	Yes	Yes	Yes	Yes	No	No
No	No	No	No	No	Yes	Yes	Yes	Yes	No	No
Yes	Yes	Yes	Yes	No	Yes	Yes	Yes	Yes	No	No
Yes	Yes	Yes	Yes	No	Yes	Yes	Yes	Yes	Yes	No
Yes	Yes	Yes	Yes	No	Yes	Yes	Yes	No	Yes	Yes
No	Yes	No	No	No	Yes	Yes	Yes	Yes	No	No
Yes	Yes	Yes	Yes	No	Yes	Yes	Yes	Yes	No	No
Yes	No	No	No	No	Yes	Yes	No	No	No	No
No	No	No	No	No	Yes	Yes	No	Yes	No	No
No	No	No	No	No	Yes	Yes	No	Yes	No	No

Supplementary Table 1

Yes	Yes	Yes	No	No	Yes	Yes	Yes	Yes	No	Yes
Yes	Yes	Yes	Yes	No	Yes	Yes	Yes	Yes	No	No
No	No	No	No	No	Yes	Yes	Yes	Yes	No	No
No	No	No	No	No	Yes	Yes	Yes	No	No	No
No	No	No	No	No	Yes	No	Yes	Yes	No	No
Yes	No	No	No	No	Yes	Yes	Yes	Yes	No	No
No	Yes	No	No	No	Yes	Yes	No	No	No	No
Yes	Yes	No	No	No	Yes	Yes	Yes	Yes	Yes	No
Yes	Yes	No	No	No	Yes	No	No	Yes	No	No
Yes	Yes	No	No	No	Yes	No	Yes	No	No	No
No	No	No	No	No	Yes	Yes	No	Yes	No	No
Yes	No	No	No	No	Yes	Yes	No	Yes	No	No
Yes	No	No	No	No	Yes	Yes	No	Yes	No	No
No	Yes	Yes	No	No	Yes	Yes	No	No	No	No
Yes	Yes	No	No	No	Yes	No	No	Yes	No	No
No	Yes	No	No	No	Yes	Yes	Yes	No	No	No
No	No	No	No	No	Yes	Yes	No	Yes	No	No

Supplementary Table 1

Yes	Yes	Yes	Yes	No	Yes	No	Yes	Yes	No	No
Yes	Yes	Yes	Yes	No	Yes	Yes	Yes	Yes	Yes	Yes
Yes	Yes	Yes	Yes	No	Yes	Yes	Yes	No	No	No
No	No	No	No	No	No	No	No	No	No	No
Yes	No	No	No	No	Yes	Yes	Yes	Yes	No	No
No	No	No	No	No	No	No	No	No	No	No
Yes	Yes	No	No	No	Yes	Yes	No	Yes	No	No
Yes	No	No	No	No	Yes	No	No	Yes	No	No
Yes	No	No	No	No	Yes	No	No	No	No	No
Yes	No	No	No	No	Yes	Yes	No	Yes	No	No
No	No	No	No	No	No	No	No	No	No	No
Yes	No	No	No	No	Yes	Yes	Yes	Yes	Yes	No
No	Yes	Yes	Yes	No	Yes	Yes	Yes	Yes	No	No
Yes	Yes	No	No	No	Yes	Yes	Yes	Yes	No	No
Yes	No	No	No	No	Yes	Yes	Yes	No	No	No

Supplementary Table 1

Yes	No	No	No	No	Yes	No	Yes	Yes	No	No
Yes	Yes	Yes	Yes	No	Yes	No	No	Yes	No	No
Yes	No	No	No	No	Yes	No	Yes	Yes	No	No
Yes	No	No	No	No	Yes	No	Yes	No	No	No
No	Yes	No	No	No	Yes	No	Yes	Yes	No	No
Yes	No	No	No	No	Yes	Yes	Yes	Yes	No	No
Yes	No	No	No	No	Yes	Yes	Yes	Yes	No	No
No	No	No	No	No	No	No	No	No	No	No
No	No	No	No	No	Yes	Yes	No	No	No	No
Yes	Yes	Yes	Yes	No	Yes	Yes	Yes	No	No	Yes
No	Yes	No	No	No	Yes	No	No	No	No	No
No	Yes	Yes	Yes	No	Yes	Yes	Yes	Yes	No	No
Yes	Yes	Yes	Yes	No	Yes	Yes	Yes	Yes	No	No
Yes	Yes	Yes	Yes	No	Yes	No	Yes	Yes	No	No
Yes	No	No	No	No	Yes	No	Yes	No	No	No
Yes	Yes	Yes	Yes	No	Yes	Yes	Yes	Yes	No	No
No	No	No	No	No	Yes	Yes	No	No	No	No

Supplementary Table 1

Yes	Yes	Yes	Yes	No	Yes	Yes	Yes	No	No	No
Yes	No	No	No	No	Yes	No	No	No	No	No
Yes	Yes	No	No	No	Yes	No	Yes	Yes	Yes	No
No	No	No	No	No	Yes	No	No	Yes	Yes	Yes
Yes	No	No	No	No	Yes	Yes	Yes	Yes	No	No
Yes	No	No	No	No	Yes	No	Yes	Yes	No	No
No	No	No	No	No	Yes	No	No	No	No	No
Yes	No	No	No	No	Yes	No	No	No	No	No
Yes	Yes	Yes	Yes	No	Yes	Yes	Yes	No	No	No
Yes	Yes	Yes	Yes	No	Yes	No	No	No	No	No
No	No	No	No	No	Yes	No	No	No	No	No
No	No	No	No	No	Yes	No	No	No	No	No
No	No	No	No	No	Yes	No	No	No	No	No
No	No	No	No	No	Yes	No	No	No	No	No
No	No	No	No	No	No	No	No	No	No	No

Supplementary Table 1

No	No	No	No	No	Yes	Yes	No	No	No	No
Yes	Yes	No	No	No	Yes	Yes	No	No	No	No
Yes	Yes	No	No	No	Yes	No	No	No	No	No
No	No	No	No	No	Yes	No	No	No	No	No
No	No	No	No	No	Yes	No	No	No	No	No
No	No	No	No	No	Yes	No	No	No	No	No
No	No	No	No	No	Yes	Yes	No	No	No	No
No	No	No	No	No	Yes	No	No	No	No	No
No	No	No	No	No	Yes	Yes	Yes	Yes	No	No
Yes	No	No	No	No	Yes	Yes	Yes	Yes	No	No
No	No	No	No	No	No	No	No	No	No	No
No	No	No	No	No	Yes	No	No	No	No	No
No	No	No	No	No	Yes	No	No	No	No	No
No	No	No	No	No	Yes	No	No	No	No	No
No	No	No	No	No	Yes	No	Yes	No	No	No
No	No	No	No	No	Yes	No	No	No	No	No
No	No	No	No	No	Yes	No	No	No	No	No

Supplementary Table 1

No	No	No	No	No	Yes	No	No	No	No	No
No	No	No	No	No	Yes	No	No	No	No	No
No	No	No	No	No	Yes	No	No	No	No	No
No	No	No	No	No	Yes	No	No	No	No	No
Yes	No	No	No	No	Yes	No	No	No	No	No
No	No	No	No	No	Yes	No	No	No	No	No
No	No	No	No	No	Yes	No	No	No	No	No
No	No	No	No	No	Yes	No	No	No	No	No
No	No	No	No	No	Yes	No	No	Yes	No	No
No	No	No	No	No	Yes	Yes	No	Yes	No	No
No	No	No	No	No	Yes	No	No	No	No	No
Yes	No	No	No	No	Yes	No	No	No	No	No
Yes	No	No	No	No	Yes	No	No	No	No	No
No	No	No	No	No	Yes	No	No	No	No	No
Yes	Yes	No	No	No	Yes	No	No	No	No	No
No	No	No	No	No	Yes	No	No	No	No	No
Yes	Yes	Yes	No	No	Yes	No	No	Yes	No	No

Supplementary Table 1

No	No	No	No	No	No	No	No	No	No	No
No	No	No	No	No	No	No	No	No	No	No
Yes	No	No	No	No	No	No	No	No	No	No
No	No	No	No	No	No	No	No	No	No	No
No	No	No	No	No	No	No	No	No	No	No
No	No	No	No	No	No	No	No	No	No	No
No	No	No	No	No	No	No	No	No	No	No
No	No	No	No	No	No	No	No	No	No	No
No	No	No	No	No	No	No	No	No	No	No
No	No	No	No	No	No	No	No	No	No	No
No	No	No	No	No	No	No	No	No	No	No
No	No	No	No	No	No	No	No	No	No	No
No	Yes	No	No	No	No	No	No	No	No	No
Unknown	Unknown	Unknown	Unknown	Unknown	Unknown	Unknown	Unknown	Unknown	Unknown	Unknown
Unknown	Unknown	Unknown	Unknown	Unknown	Unknown	Unknown	Unknown	Unknown	Unknown	Unknown
Unknown	Unknown	Unknown	Unknown	Unknown	Unknown	Unknown	Unknown	Unknown	Unknown	Unknown

Supplementary Table 1

No	No	No	No	No	No	No	No	No	No	No
No	No	No	No	No	No	No	No	No	No	No
Yes	Yes	Yes	No	Yes	Yes	Yes	Yes	Yes	No	No
Yes	No	No	No	No	No	No	Yes	Yes	No	No
Yes	No	No	No	No	Yes	No	No	No	No	No
No	No	No	No	No	No	Yes	Yes	No	No	No
Yes	No	No	No	No	No	No	No	No	No	No
No	No	No	No	No	No	No	No	No	No	No
No	No	No	No	No	No	No	No	No	No	No
No	No	No	No	No	No	No	No	No	No	No
Yes	Yes	Yes	No	No	Yes	Yes	Yes	Yes	Yes	Yes
No	Yes	No	No	No	No	No	No	No	No	No
Yes	Yes	Yes	No	No	No	No	No	No	No	No
Yes	Yes	Yes	No	No	No	No	No	No	No	No
Yes	Yes	Yes	Yes	No	No	No	No	No	No	No
No	Yes	No	No	No	No	No	No	No	Yes	Yes
Yes	No	No	No	No	No	No	No	No	No	No

Supplementary Table 1

Yes	No	No	No	No	No	No	No	No	No	No
Yes	Yes	No	No	No	No	No	No	No	No	No
Yes	Yes	Yes	No	No	No	No	No	No	No	No
No	No	No	No	No	No	No	No	No	No	No
Yes	Yes	No	No	No	Yes	No	Yes	Yes	No	No
Yes	Yes	No	No	No	Yes	Yes	Yes	No	No	No
Yes	Yes	Yes	No	No	Yes	No	No	Yes	No	No
Yes	Yes	Yes	No	No	Yes	No	No	Yes	No	No
No	No	No	No	No	No	No	No	No	No	No
No	Yes	No	No	No	No	No	No	No	No	No
Yes	Yes	Yes	No	No	Yes	No	No	No	No	No
Yes	Yes	No	No	No	No	No	No	No	No	No
Yes	Yes	No	No	No	No	No	No	No	No	No
Yes	Yes	Yes	Yes	Yes	Yes	Yes	Yes	Yes	Yes	Yes
Yes	Yes	Yes	Yes	No	Yes	No	No	No	No	No
Yes	Yes	Yes	Yes	No	Yes	Yes	Yes	Yes	No	No

Supplementary Table 1

Yes	Yes	Yes	Yes	Yes	Yes	Yes	Yes	Yes	No	No
Yes	Yes	Yes	Yes	No	Yes	Yes	Yes	Yes	Yes	Yes
No	No	No	No	No	Yes	No	No	No	No	No
No	Yes	Yes	Yes	No	Yes	Yes	Yes	Yes	Yes	No
No	No	No	No	No	Yes	Yes	Yes	No	No	No
No	Yes	Yes	Yes	No	Yes	Yes	Yes	Yes	No	No
No	Yes	Yes	Yes	No	Yes	Yes	Yes	No	No	No
Yes	No	No	No	No	Yes	Yes	Yes	Yes	No	No
No	No	No	No	No	Yes	Yes	Yes	Yes	No	No
Yes	No	No	No	No	Yes	Yes	Yes	No	No	No
Yes	Yes	Yes	Yes	No	Yes	Yes	No	No	No	No
Yes	Yes	Yes	Yes	No	Yes	Yes	No	No	No	No
No	Yes	Yes	Yes	No	Yes	Yes	Yes	Yes	No	No
Yes	No	No	No	No	Yes	No	Yes	Yes	No	No
No	No	No	No	No	Yes	No	Yes	Yes	No	No
No	No	No	No	No	No	No	No	Yes	No	No

Supplementary Table 1

No	No	No	No	No	Yes	Yes	Yes	No	No	No
No	Yes	Yes	No	No	Yes	Yes	Yes	No	No	No
Yes	Yes	No	No	No	Yes	Yes	Yes	Yes	No	No
Yes	Yes	Yes	No	No	Yes	No	No	No	No	No
No	Yes	No	No	No	Yes	Yes	No	No	No	No
No	No	No	No	No	No	No	No	No	No	No
Yes	Yes	No	No	No	Yes	Yes	Yes	Yes	Yes	No
Yes	No	No	No	No	Yes	Yes	Yes	No	No	No
Yes	No	No	No	No	Yes	No	Yes	No	No	No
Yes	No	No	No	No	Yes	Yes	Yes	No	No	No
Yes	Yes	Yes	No	No	Yes	No	Yes	No	Yes	Yes
Yes	Yes	Yes	Yes	No	Yes	No	Yes	No	Yes	Yes
Yes	Yes	Yes	Yes	No	Yes	No	Yes	Yes	No	No
Yes	No	No	No	No	Yes	No	Yes	Yes	No	No
Yes	No	No	No	No	Yes	No	No	No	No	No
Yes	No	No	No	No	No	No	No	No	No	No
Yes	No	No	No	No	Yes	No	No	No	No	No
Yes	No	No	No	No	Yes	No	No	No	No	No
Yes	No	No	No	No	Yes	No	No	No	No	No
Yes	No	No	No	No	Yes	No	No	No	No	No
Yes	Yes	No	No	No	No	No	No	No	No	No
Yes	No	No	No	No	Yes	No	Yes	No	No	No
No	No	No	No	No	No	No	No	No	No	No
Yes	No	No	No	No	No	No	No	No	No	No
No	No	No	No	No	No	No	No	No	No	No
No	No	No	No	No	No	No	No	No	No	No

Supplementary Table 1

Yes	No	No	No	No	No	No	No	No	No	No
Yes	No	No	No	No	No	No	No	No	No	No
Yes	No	No	No	No	No	No	No	No	No	No
Yes	No	No	No	No	Yes	Yes	Yes	No	No	No
Yes	No	No	No	No	Yes	No	No	No	No	No
Yes	No	No	No	No	No	No	No	No	No	No
Yes	No	No	No	No	Yes	Yes	Yes	No	No	No
No	No	No	No	No	No	No	No	No	No	No
Yes	Yes	No	No	No	Yes	No	Yes	Yes	Yes	No
Yes	No	No	No	No	Yes	No	No	No	No	No
No	No	No	No	No	No	No	No	No	No	No
Yes	Yes	Yes	Yes	No	No	No	No	Yes	Yes	No
Yes	Yes	Yes	Yes	No	Yes	Yes	Yes	Yes	Yes	No
Yes	Yes	Yes	Yes	No	Yes	No	Yes	Yes	No	No
No	No	No	No	No	No	No	No	No	No	No
No	No	No	No	No	No	No	No	No	No	No
No	No	No	No	No	No	No	No	No	No	No
No	No	No	No	No	No	No	No	No	No	No
No	Yes	Yes	Yes	No	No	No	No	No	No	No

Supplementary Table 1

Yes	Yes	Yes	Yes	No	Yes	No	Yes	Yes	Yes	No
No	No	No	No	No	No	No	No	No	No	No
No	No	No	No	No	No	No	No	No	No	No
Yes	Yes	Yes	Yes	No	Yes	No	No	No	No	No
No	No	No	No	No	No	No	No	No	No	No
Yes	No	No	No	No	Yes	No	Yes	No	No	No
No	No	No	No	No	No	No	No	No	No	No
Yes	No	No	No	No	Yes	No	No	No	No	No
No	No	No	No	No	No	No	No	No	No	No
Yes	Yes	No	No	No	Yes	No	Yes	Yes	No	No
No	No	No	No	No	No	No	No	No	No	No
No	No	No	No	No	No	No	No	No	No	No
-	-	-	-	-	-	-	-	-	-	-
No	No	No	No	No	No	No	No	No	No	No

Supplementary Table 1

Yes	No	No	No	No	Yes	No	No	No	No	No
No	No	No	No	No	No	No	No	No	No	No
Yes	No	No	No	No	Yes	No	Yes	Yes	No	No
No	No	No	No	No	No	No	No	No	No	No
Yes	Yes	Yes	Yes	No	Yes	No	Yes	No	No	No
No	No	No	No	No	No	No	No	No	No	No
No	No	No	No	No	No	No	No	No	No	No
No	No	No	No	No	No	No	No	No	No	No
Yes	No	No	No	No	No	No	No	No	No	No
No	No	No	No	No	No	No	No	No	No	No
No	No	No	No	No	No	No	No	No	No	No
No	No	No	No	No	No	No	No	No	No	No
Yes	No	No	No	No	No	No	No	No	No	No

Supplementary Table 1

Yes	No	No	No	No	Yes	Yes	Yes	No	No	No
Yes	Yes	No	No	No	No	No	No	No	No	No
Yes	No	No	No	No	Yes	Yes	Yes	Yes	Yes	No
No	No	No	No	No	No	No	No	No	No	No
Yes	Yes	Yes	Yes	No	Yes	No	Yes	No	No	No
Yes	Yes	No	No	No	Yes	Yes	Yes	Yes	No	No
Yes	Yes	No	No	No	Yes	No	No	Yes	No	No
No	No	No	No	No	No	No	No	No	No	No
No	Yes	Yes	No	No	No	No	No	No	No	No
No	Yes	No	No	No	No	No	No	Yes	No	No
No	No	No	No	No	No	No	No	No	No	No
No	No	No	No	No	No	No	No	No	No	No
No	Yes	Yes	No	No	Yes	No	No	Yes	No	No
No	No	No	No	No	No	No	No	No	No	No
Yes	Yes	Yes	No	No	No	No	No	Yes	No	No
No	No	No	No	No	No	No	No	No	No	No
Yes	No	No	No	No	No	No	No	No	No	No
Yes	Yes	Yes	No	No	Yes	No	No	No	No	No
No	No	No	No	No	No	No	No	No	No	No

Supplementary Table 1

No	No	No	No	No	No	No	No	No	No	No
Yes	No	No	No	No	No	No	No	No	No	No
Yes	Yes	No	No	No	Yes	No	No	No	No	No
Yes	Yes	No	No	No	Yes	Yes	Yes	Yes	No	No
No	No	No	No	No	No	No	No	No	No	No
No	No	No	No	No	No	No	No	No	No	No
Yes	No	Yes	No	No	Yes	No	No	No	No	No
Yes	Yes	Yes	No	No	No	No	No	No	No	No
No	No	No	No	No	No	No	No	No	No	No
No	No	No	No	No	No	No	No	No	No	No
No	Yes	No	No	No	No	No	No	No	No	No
No	No	No	No	No	No	No	No	No	No	No
Yes	No	No	No	No	Yes	Yes	Yes	Yes	No	No
No	No	No	No	No	No	No	No	No	No	No
No	No	No	No	No	No	No	No	No	No	No
No	No	No	No	No	No	No	No	No	No	No
Yes	Yes	No	No	No	Yes	Yes	Yes	Yes	Yes	Yes
No	No	No	No	No	No	No	No	No	No	No
No	No	No	No	No	No	No	No	No	No	No
No	No	No	No	No	No	No	No	No	No	No

Supplementary Table 1

Yes	No	No	No	No	No	No	No	No	No	No
Yes	Yes	No	No	No	Yes	Yes	Yes	Yes	Yes	Yes
No	No	No	No	No	No	No	No	No	No	No
No	No	No	No	No	No	No	No	No	No	No
No	No	No	No	No	No	No	No	No	No	No
Yes	No	No	No	No	No	No	No	Yes	No	No
No	No	No	No	No	No	No	No	No	No	No
No	No	No	No	No	No	No	No	No	No	No
