# Supplementary material for: Vaccinology in sub-Saharan Africa
Source: BMJ Glob Health. 2019 Sep 20;4(5):e001363. doi: 10.1136/bmjgh-2018-001363 (PMC6768329; doi:10.1136/bmjgh-2018-001363)
Supplement: Supplementary data [file bmjgh-2018-001363supp005.pdf]

Supplementary Table 2

Vaccine-preventable disease literature search results: number of papers meeting search criteria, Jan 1st 2010-Sep 30th 2016

Country	Diptheria	Tetanus	Pertussis	Tuberculosis	TB	Poliomyelitis	Polio	Hinfluenzab	Hib	Hepb
Angola	1	2	1	15	8	12	18	2	1	6
Benin	4	7	2	65	30	1	2	0	1	23
Botswana	0	1	1	100	57	1	2	0	0	13
Burkina Faso	2	8	3	58	27	1	5	3	1	25
Burundi	0	2	1	14	4	1	2	0	0	3
Cameroon	5	13	6	152	79	11	14	2	3	57
Cape Verde	0	0	0	0	0	0	0	0	0	0
Central African Republic	0	0	0	21	9	3	4	0	0	12
Chad	2	5	2	16	6	15	20	0	0	7
Comoros	0	1	0	2	1711	0	0	0	0	3
Congo	8	15	8	93	46	63	81	1	1	33
DR Congo	0	7	2	56	29	22	28	0	0	14
Equatorial Guinea	0	0	0	2	0	2	2	0	0	2
Eritrea	0	0	0	14	8	0	0	0	0	2
Ethiopia	8	30	9	535	415	18	28	1	4	57
Gabon	4	4	4	35	15	4	8	2	1	19
Gambia	8	11	9	101	59	0	5	11	10	35
Ghana	11	21	13	154	91	7	19	3	2	61
Guinea	48	55	50	345	148	15	40	8	7	27
Guinea Bissau	35	35	35	49	27	1	18	5	4	10
Ivory Coast	4	8	4	88	38	3	7	0	0	24
Kenya	11	29	9	273	176	19	35	4	7	39
Lesotho	0	0	0	20	23	0	0	0	0	2
Liberia	0	2	0	14	6	3	7	0	0	2
Madagascar	2	3	2	42	19	14	15	0	0	8
Malawi	0	1	0	216	129	2	3	4	1	15

Supplementary Table 2

Mali	6	16	5	44	19	2	4	9	8	11
Mauritania	1	1	1	4	1	1	0	0	0	2
Mauritius	0	0	0	4	0	0	0	0	0	2
Mozambique	5	7	5	83	40	3	7	4	5	14
Namibia	0	1	0	51	29	2	2	0	0	3
Niger	3	14	5	93	51	4	6	4	3	45
Nigeria	24	74	24	381	217	194	247	5	5	183
Rwanda	0	1	0	58	27	1	4	1	0	17
Sao Tome and Principe	0	0	0	0	0	0	0	0	0	0
Senegal	5	14	7	61	18	0	5	6	5	0
Seychelles	0	0	0	0	0	0	0	0	0	0
Sierra Leone	0	0	2	24	5	4	9	0	0	3
Somalia	4	6	4	47	27	21	24	0	0	11
South Africa	29	57	42	2885	1893	32	55	16	19	257
South Sudan	0	1	0	12	5	3	3	0	0	7
Sudan	0	4	0	80	38	7	8	0	0	32
Swaziland	0	0	0	39	28	0	0	0	0	0
Tanzania	8	21	9	313	200	5	15	6	2	32
Togo	0	2	0	32	12	3	7	0	0	14
Uganda	3	15	4	437	333	4	18	3	2	40
Zambia	3	6	3	179	123	2	4	0	0	15
Zimbabwe	5	4	3	102	79	5	11	0	0	8

Supplementary Table 2

Yellow fever	Measles	Rubella	S. pneumoniae	Pneumococcus	Rotavirus	H. papillomavirus	HPV	N. meningitidis	Meningococcus	Typhoid	Dengue
18	4	0	3	3	2	0	0	2	2	1	19
3	5	3	2	2	2	8	10	3	4	9	7
0	5	1	1	1	7	5	12	0	0	0	0
4	13	3	15	15	13	7	12	39	44	8	8
0	4	0	0	0	0	0	0	0	0	0	0
11	16	1	6	6	20	10	19	4	5	9	16
0	0	0	0	0	0	0	0	0	0	0	0
7	8	6	2	2	5	0	2	0	2	0	10
0	5	0	1	1	0	0	0	6	6	3	0
0	1	0	0	0	0	0	1	0	0	3	11
23	47	9	7	7	37	11	15	2	2	8	42
8	30	8	5	5	10	5	10	0	0	8	2
0	0	0	0	0	0	0	0	0	0	0	0
0	4	0	0	0	0	0	0	0	0	0	0
6	38	5	17	17	12	7	14	8	8	9	4
6	6	4	7	7	2	0	6	0	0	4	24
4	16	0	45	48	4	0	0	7	10	0	0
10	16	0	22	24	49	7	12	9	11	17	7
13	52	7	32	33	22	7	12	4	3	18	43
3	43	3	1	1	4	0	0	0	0	6	1
15	11	5	3	3	3	3	5	3	3	1	10
24	47	7	33	35	46	31	0	3	3	31	38
0	3	0	0	0	0	0	2	0	0	0	0
2	5	0	0	0	0	2	2	0	0	3	3
2	9	0	4	5	4	2	8	0	0	8	17
0	1	1	34	38	36	1	11	2	2	16	0

Supplementary Table 2

9	5	0	11	11	10	4	8	20	23	5	4
2	2	0	0	0	0	0	1	0	0	0	2
0	0	0	0	0	2	0	0	0	0	0	13
3	9	2	8	9	4	7	11	2	2	5	7
0	7	2	1	1	1	1	1	1	1	0	1
2	15	2	20	21	8	12	8	31	33	8	2
15	66	17	27	28	17	48	64	12	15	57	41
0	8	3	3	4	13	6	19	0	0	0	1
2	0	0	0	0	1	2	0	0	0	0	2
21	10	0	11	13	6	8	10	12	13	7	24
0	0	0	0	0	0	0	0	0	0	0	6
9	12	0	0	0	0	0	0	0	0	5	6
0	14	4	0	2	0	0	2	0	0	0	3
85	87	21	93	101	117	114	197	35	43	49	93
4	5	0	0	0	1	0	0	0	0	3	5
14	0	4	0	0	5	17	15	0	0	3	18
0	2	0	0	0	0	0	0	0	0	0	0
9	25	8	20	20	20	16	42	1	1	28	27
2	4	0	3	3	2	0	0	7	7	5	0
23	32	5	13	13	9	42	66	3	3	11	26
4	9	1	2	2	19	6	13	0	1	6	3
3	14	4	1	1	5	9	15	0	0	5	1

Supplementary Table 2

Cholera	Vaccine Preventable Disease	Vaccines	Vaccination	Immunization	Deduptotal	Pubrate	Pop 2015	Sub-region
9	1	34	20	34	106	4,235058	25029174	Central
4	3	45	34	51	156	14,33846	10879829	Western
0	0	15	17	21	127	56,13296884	2262485	Southern
2	6	105	67	92	244	13,47651579	18105570	Western
2	0	6	8	8	30	2,683622149	11178921	Eastern
18	7	87	83	111	367	15,72126	23344179	Central
0	0	0	0	0	0	0	520502	Western
5	2	16	24	29	84	17,1418986	4900274	Central
4	0	33	35	44	82	5,841507645	14037472	Central
4	0	8	8	8	36	45,65781497	788474	Eastern
35	12	182	122	194	222	48,04852	4620330	Central
30	6	68	55	78	222	2,873161044	77266814	Central
0	0	6	0	0	10	11,83347928	845060	Central
0	0	5	5	5	22	4,59331614	4789568	Eastern
8	13	122	138	185	637	6,40904712	99390750	Eastern
4	0	36	25	32	128	74,19033995	1725292	Central
9	6	175	102	132	252	126,5743946	1990924	Western
16	18	132	90	141	386	14,08250715	27409892	Western
53	11	715	339	520	565	44,81072	12608590	Western
5	0	109	74	79	155	84,04158703	1844325	Western
6	1	33	46	46	137	6,034828626	22701556	Western
42	17	342	268	368	749	16,26482276	46050302	Eastern
0	2	4	5	6	30	14,05137746	2135022	Southern
0	2	28	18	29	70	15,54368	4503438	Western
2	2	46	32	37	158	6,519391683	24235390	Eastern
7	6	102	77	98	354	20,56318497	17215232	Eastern

Supplementary Table 2

16	6	130	85	109	214	12,15930232	17599694	Western
0	0	5	5	7	9	2,212626	4067564	Western
3	0	7	2	3	33	26,13644014	1262605	Eastern
26	5	54	44	61	161	5,754549588	27977863	Eastern
0	1	13	14	22	19	7,727252392	2458830	Southern
2	8	60	62	89	248	12,46286268	19899120	Western
23	42	366	341	549	1023	5,61464865	182201962	Western
2	2	33	38	52	174	14,98751127	11609666	Eastern
0	0	1	1	2	7	36,77552221	190344	Central
8	5	92	60	83	305	20,15959392	15129273	Western
0	0	0	0	0	6	64,58557589	92900	Eastern
9	2	49	33	43	104	16,11608	6453184	Western
0	0	28	29	39	217	20,11661332	10787104	Eastern
64	159	1077	694	907	2931	53,33268313	54956920	Southern
10	3	20	18	24	62	5,024388	12339812	Eastern
16	4	86	68	94	194	4,821686814	40234882	Eastern
0	0	2	3	5	44	34,18883113	1286970	Southern
29	8	177	148	201	536	10,02423396	53470420	Eastern
5	2	14	16	23	176	24,09447883	7304578	Western
15	6	176	136	187	621	15,90986643	39032383	Eastern
6	3	51	50	66	251	15,48258126	16211767	Eastern
25	3	45	49	61	190	12,17733975	15602751	Eastern
