# Supplementary figures and images for: Vaccinology in sub-Saharan Africa
Source: BMJ Glob Health. 2019 Sep 20;4(5):e001363. doi: 10.1136/bmjgh-2018-001363 (PMC6768329; doi:10.1136/bmjgh-2018-001363)

### Number of vaccine-related scientific publications in Sub-Saharan Africa, per million people

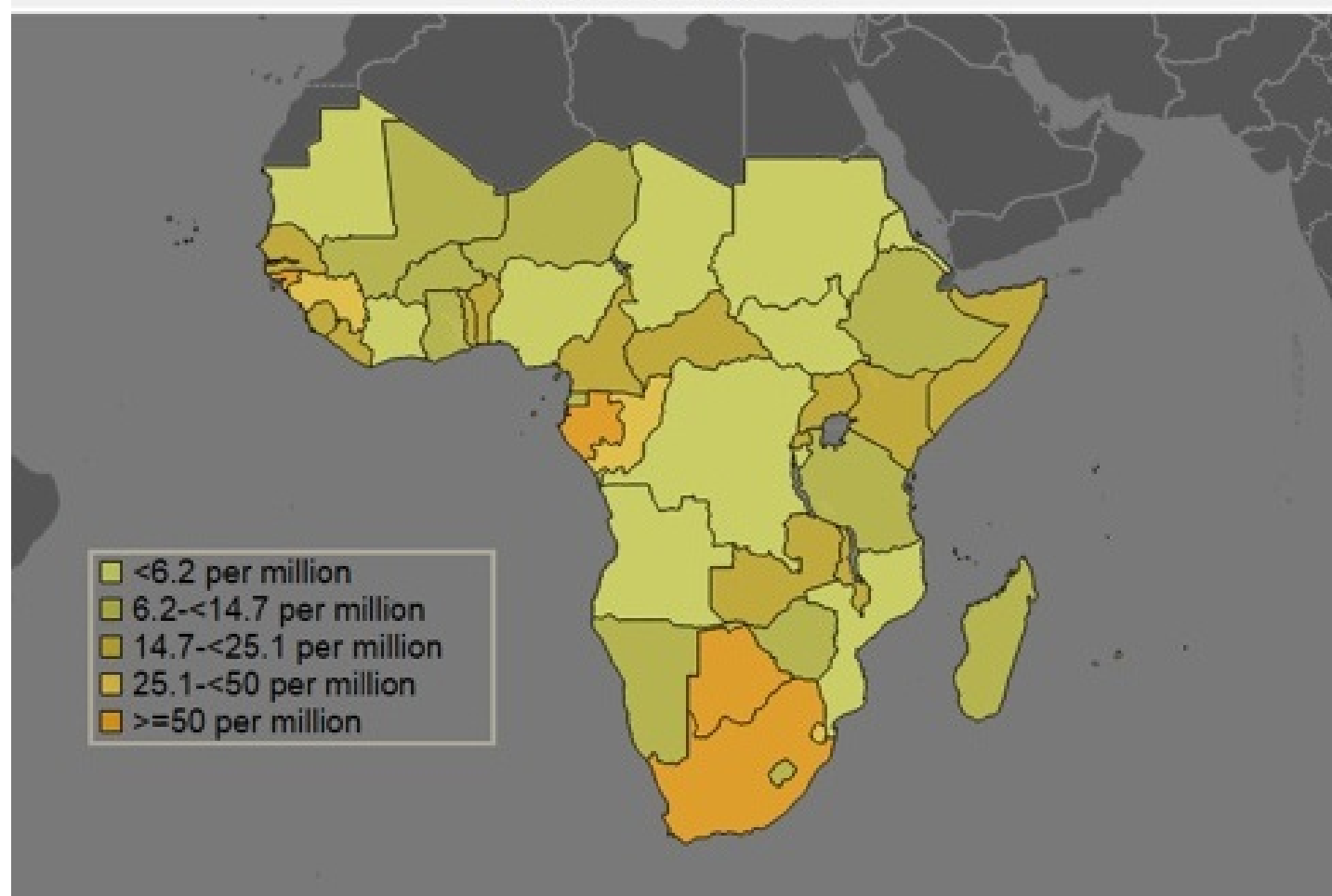

Supplement: Supplementary data [file bmjgh-2018-001363supp006.pdf]

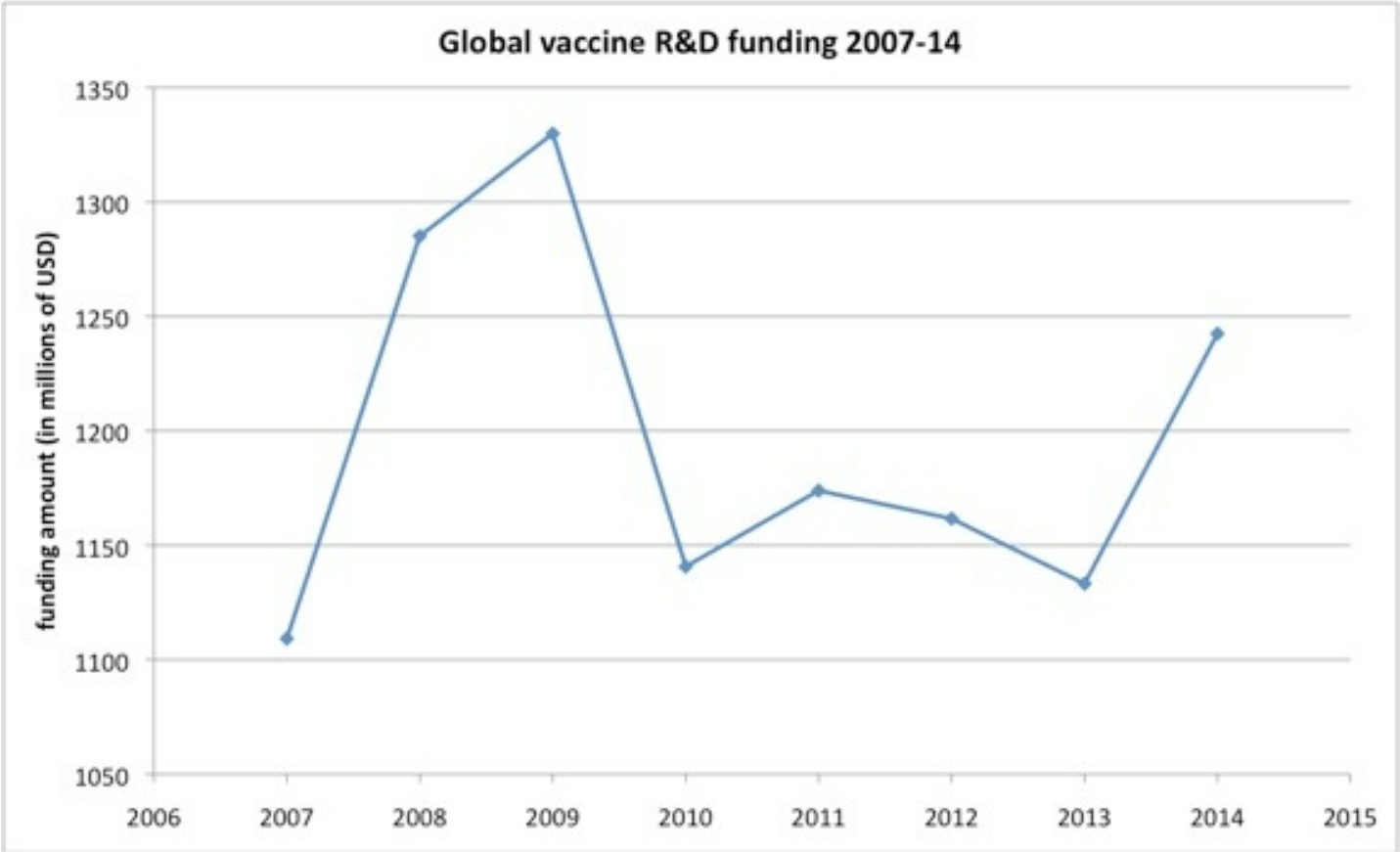

Supplement: Supplementary data [file bmjgh-2018-001363supp008.pdf]

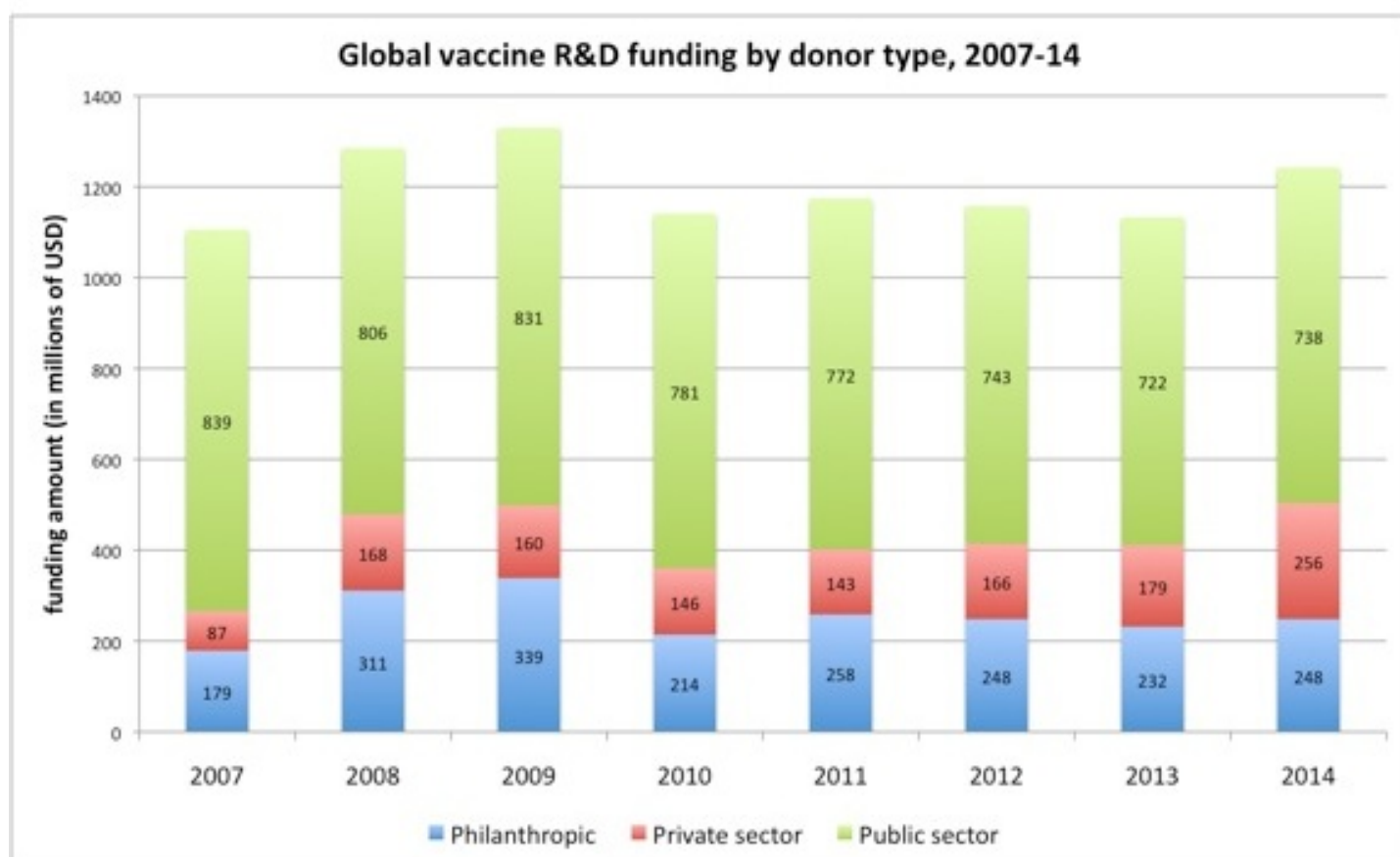

Supplement: Supplementary data [file bmjgh-2018-001363supp009.pdf]

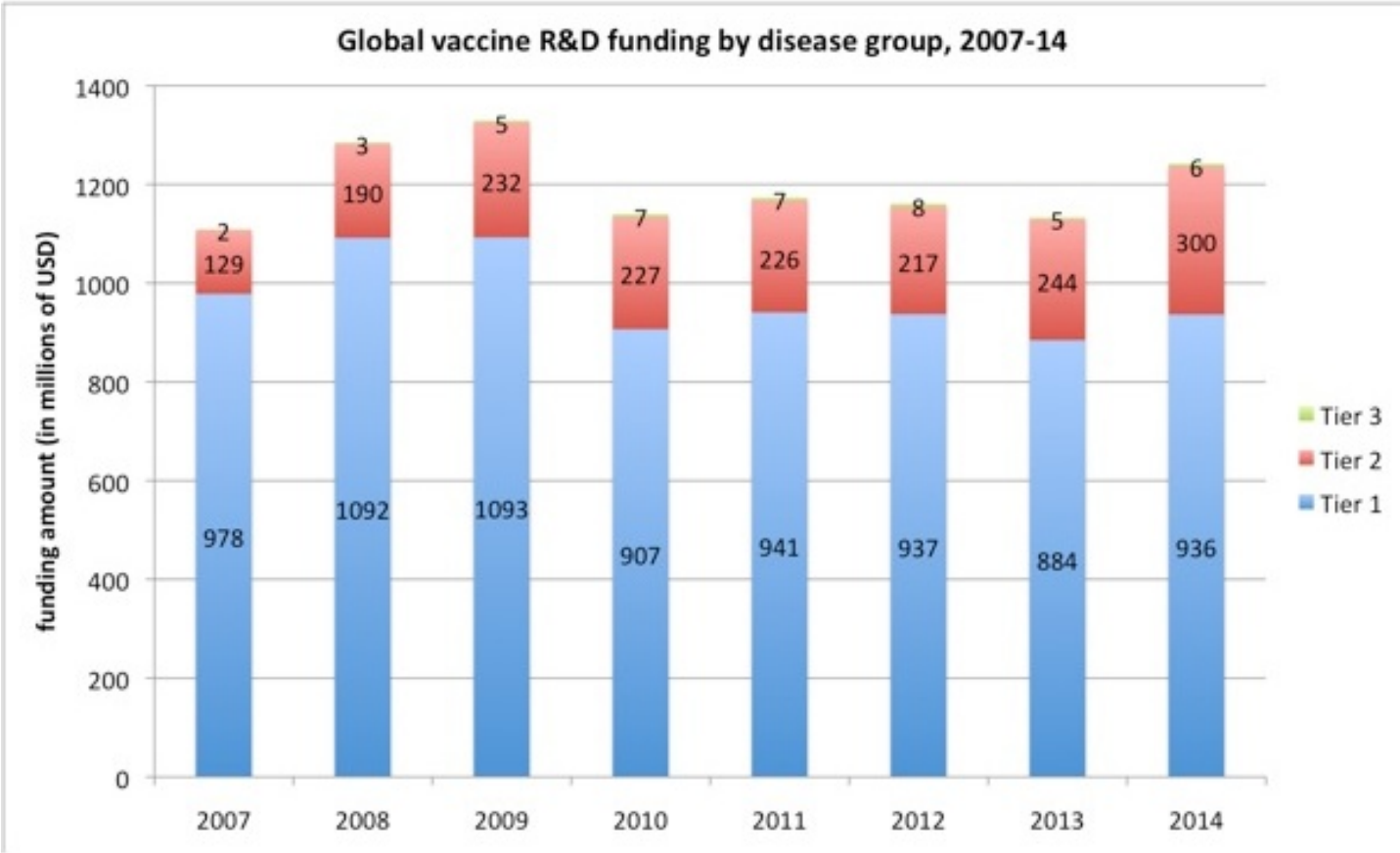

Supplement: Supplementary data [file bmjgh-2018-001363supp010.pdf]

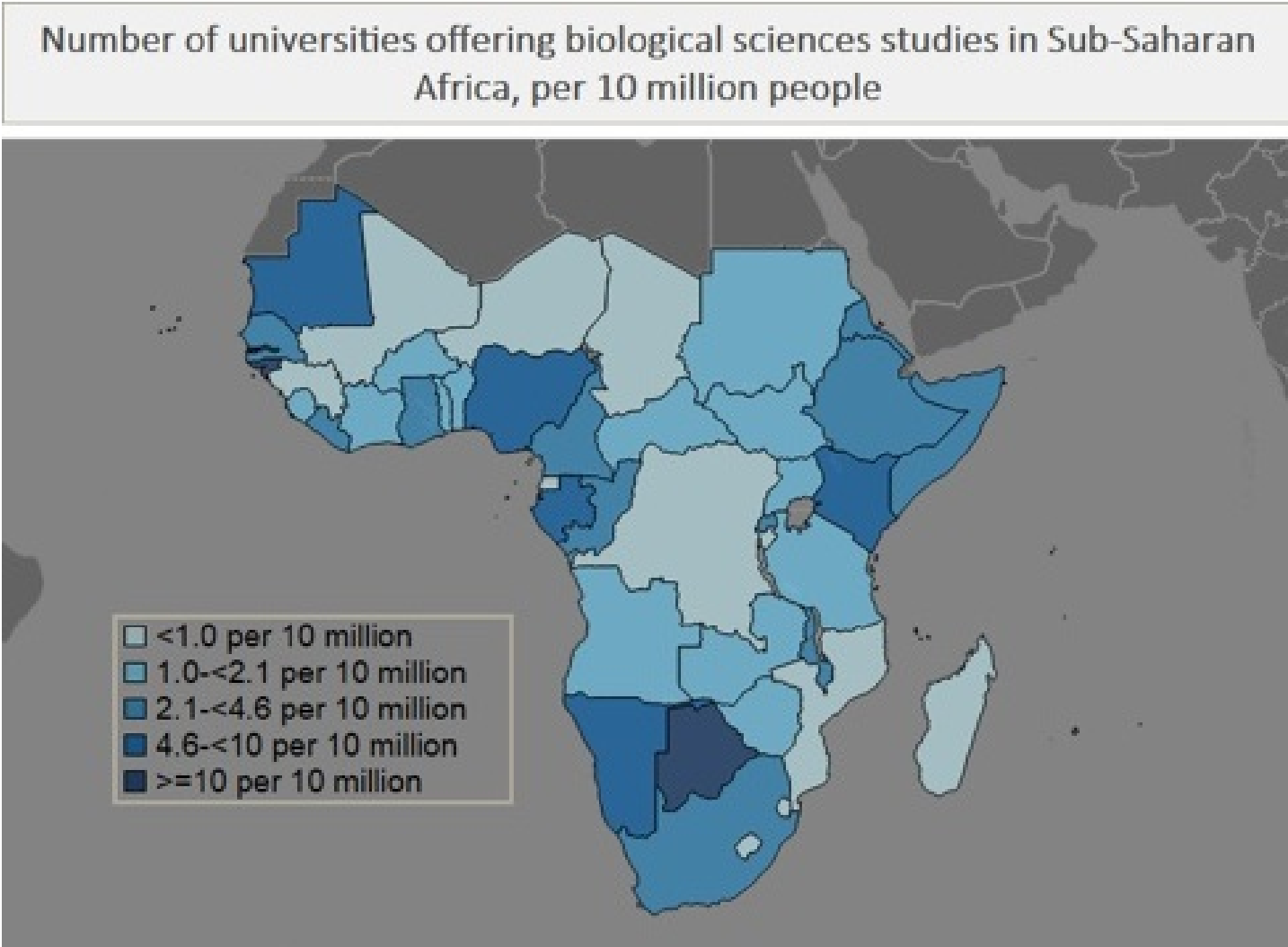

Supplement: Supplementary data [file bmjgh-2018-001363supp002.pdf]

### Number of universities offering medical studies in Sub-Saharan Africa, per 10 million people

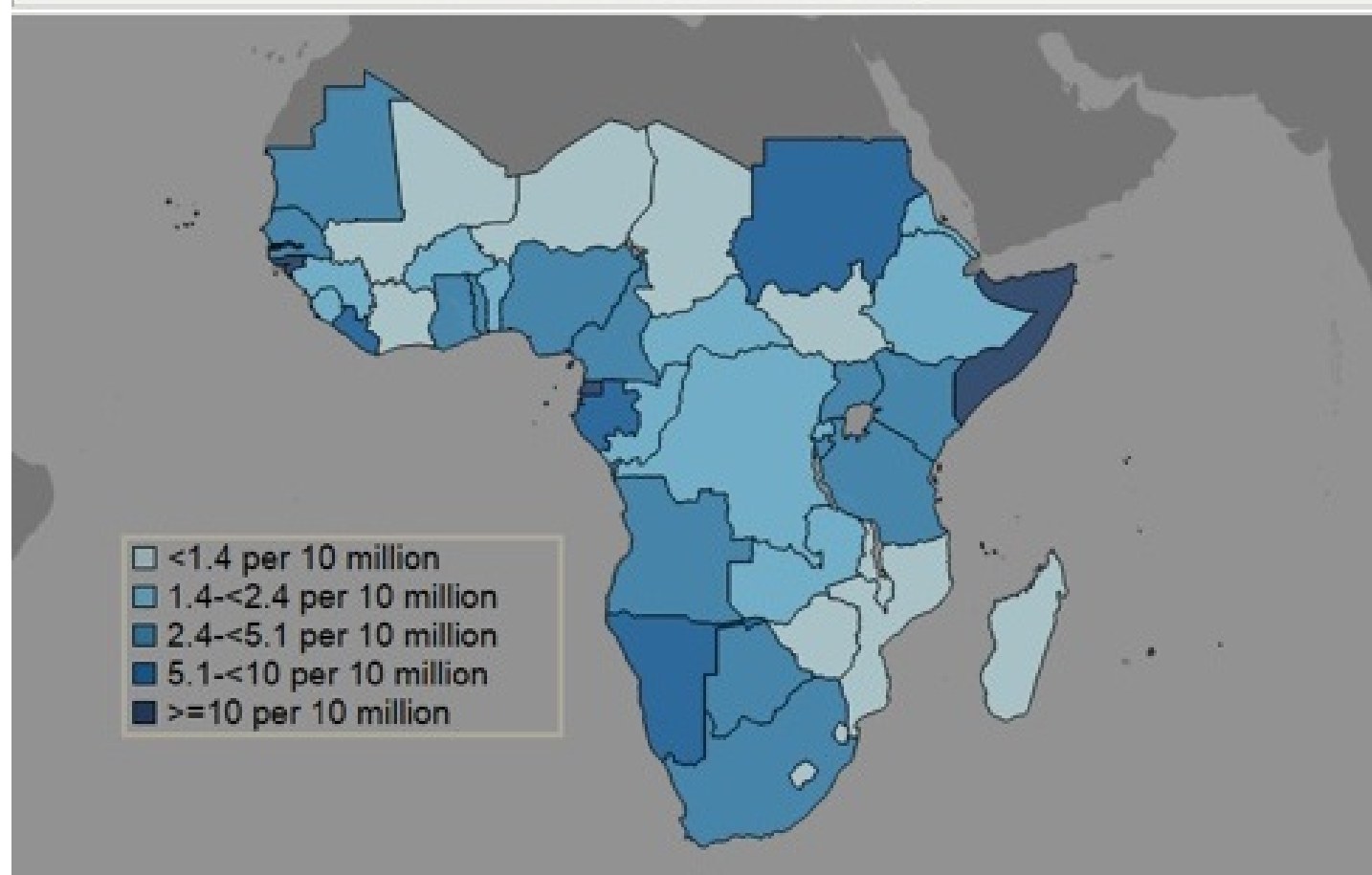

Supplement: Supplementary data [file bmjgh-2018-001363supp003.pdf]
