# Supplementary material for: Vaccinology in sub-Saharan Africa
Source: BMJ Glob Health. 2019 Sep 20;4(5):e001363. doi: 10.1136/bmjgh-2018-001363 (PMC6768329; doi:10.1136/bmjgh-2018-001363)
Supplement: Supplementary data [file bmjgh-2018-001363supp007.pdf]

Supplementary Table 3

List of vaccinology research institutions and description of capacity

Country	City	Institute Name	Non-African partner
Burkina Faso	Ouagadougou	Centre National de Recherche et de Formation sur le	
Burkina Faso	Bobo-Dioulasso	Centre Muraz	Agence de Médecine Préventive
Burkina Faso	Nouna	Centre de Recherche en Santé de Nouna	Univ of Heidelberg
Burkina Faso	Ouagadogou, Bobo-Dioulasso, Nanoro, Kaya	Institut de Recherche en Sciences de la Santé	Institute of Tropical Medicine, Antwerp, Belgium
Cameroon	Yaoundé	Centre Pasteur Cameroon	
Central African Republic	Bangui	Institut Pasteur de Bangui	
Cote d'Ivoire	Taabo	Centre Suisse de Recherches Scientifiques en Cote d'Ivoire	Swiss Tropical and Public Health Institute, LSHTM, others
Ethiopia	Harar/Kersa	Haramaya University	LSHTM
Gabon	Lambarene	Lambarene Medical Research Center, Albert Schweitzer Hospital	Univ. of Tubingen
Gambia	Basse, Farefenni, Fajara	MRC Gambia	
Ghana	Accra	Noguchi Memorial Institute for Medical Research	
Ghana	Navrongo	Navrongo Health Research Center	
Ghana	Kintampo	Kintampo Health Research Centre	LSHTM
Guinea	Conakry	Institut Pasteur de Guinee	
Guinea-Bissau	Bandim	Bandim Health Project	Univ. of Copenhagen
Ivory Coast	Abidjan	Institut Pasteur de Cote d'Ivoire	
Kenya	Kilifi and Nairobi	KEMRI/Wellcome Trust	Wellcome Trust/Univ of Oxford
Kenya	Kisumu and Nairobi	KEMRI/CDC	US CDC
Kenya	Kericho, Kisumu and Nairobi	KEMRI/Walter Reed (US MHRI)	US MHRI
Kenya	Nairobi	Africa Population and Health Research Center	

Supplementary Table 3

Kenya	Nairobi	Kenya AIDS Vaccine Initiative - Institute for Clinical Research	
Kenya	Mbita and Kwale	KEMRI/Mbita and Kwale HDSS	Nagasaki University
Madagascar	Antananarivo	Institut Pasteur de Madagascar	Institut Pasteur International
Malawi	Blantyre	Malawi-Liverpool-WT	Wellcome Trust/Univ of Liverpool
Malawi	Karonga	Karonga HDSS	LSHTM
Malawi	Lilongwe	Tidizwe Center/UNC Project-Malawi	Univ of North Carolina
Mali	Bamako	MRTC	
Mali	Bamako	CVD Mali	Univ of Maryland
Mozambique	Chokwe	Chókwè Health Research and Training Center	
Mozambique	Manhica	CISM	Barcelona International Health Research Center
Mozambique	Maputo	National Institute of Health/Polana Çanico Health Research and Training Center	US MHRI
Niger	Niamey	CERMES	
Nigeria	Abuja and Lagos	Walter Reed Project - Nigeria	US MHRI
Rwanda	Kigali	Projet San Francisco	
Senegal	Dakar	Institut Pasteur de Dakar	
Senegal	Niakhar, Mlomp, Dakar	Institut de Recherche pour le Développement	IRD-France
South Africa	Durban	Desmond Tutu HIV Research Foundation - Emavundleni Centre	
South Africa	Multiple sites	MRC - HIV prevention unit	
South Africa	Multiple sites	CAPRISA	Columbia Univ
South Africa	4 sites	The Aurum Institute	
South Africa	Shoshanguve	Setshaba Research Centre	
South Africa	Mtubatuba	Africa Health Research Institute	many
Tanzania	Ifakara, Bamagoyo, Rufiji	Ifakara Health Institute	Swiss Tropical and Public Health Institute
Tanzania	Moshi	Kilimanjaro Clinical Research Unit	
Tanzania	Mbeya	NIMR - Mbeya Medical Research Center	US MHRI
Uganda	Entebbe, Kyamulibwa	MRC/UVRI Uganda Research Center on AIDS	

Supplementary Table 3

Uganda	Kampala	Makerere University Walter Reed Project	US MHRI, UCLA
Uganda	Iganga/Mayuge	Makerere University Center for Health and Population Research/Iganga-Mayuge HDSS	
Zambia	Lusaka	Center for Infectious Disease Research in Zambia	
Zambia	Lusaka	Zambia Emory HIV Research Project	Emory Univ.
Zimbabwe	Harare	UZ-UCSF	Univ of California SF

Supplementary Table 3

Network membership	DSS	Phase 1	Phase 2	Phase 3	Phase 4	Bacteriology	Virology
	Yes	Yes	Yes (drugs)	No	No	No	No
	No	No	Yes	Yes	Yes	Yes	Yes
INDEPTH	Yes	No	no	no	no	yes	yes
INDEPTH	Yes	Yes (drugs)	Yes	Yes	Yes	Yes	Yes
Institut Pasteur International	No	No	No	No	No	Yes	Yes
Institut Pasteur International		No	No	No	No	Yes	Yes
INDEPTH	Yes	No	No	No	No	No	No
INDEPTH, CHAMPS	Yes	No	No	No	No	No	No
	no	Yes	Yes	Yes	No	Yes	No
INDEPTH	Yes	no	Yes	Yes	Yes	Yes	Yes
	no	yes	Yes	Yes	Yes (surv)	Yes	Yes
INDEPTH	Yes	yes	Yes	Yes	yes	Yes	Yes
INDEPTH	Yes	No	Yes	Yes	yes	no	no
Institut Pasteur International	No	No	No	No	No	No	No
INDEPTH	Yes	No	No	No	No	No	No
Institut Pasteur International	no	No	No	No	No	Yes	Yes
INDEPTH	Yes	Yes	Yes	Yes	Yes	Yes	Yes
INDEPTH, ACTG	yes	no	yes	yes	yes	yes	yes
HVTN, ACTG, INDEPTH	no	no	yes	yes	no	yes	yes
INDEPTH	yes	no	no	no	no	no	no

Supplementary Table 3

IAVI	no	yes	yes	no	no	yes	yes
INDEPTH	yes	no	no	no	no	no	no
	yes	no	no	no	no	yes	yes
	no	no	yes	yes	yes	yes	yes
INDEPTH	yes	no	no	no	no	no	no
HVTN	yes	no	yes	yes	yes	no	yes
	no	yes	yes	yes	no	no	no
	yes	yes	yes	yes	yes	yes	yes
INDEPTH	yes	no	no	yes	no	no	yes
INDEPTH	yes	no	yes	yes	yes	yes	yes
HVTN	no	yes	no	no	no	no	yes
Institut Pasteur International	no	no	no	no	no	yes	yes
	no	no	yes	no	no	no	yes
IAVI	no	no	no	no	no	no	yes
Institut Pasteur International	no	no	no	no	no	yes	yes
INDEPTH	yes	no	yes	yes	yes	yes	yes
	no	yes	yes	yes	no	no	yes
HVTN	no	yes	yes	yes	no	no	yes
UNAIDS collaborating center	no	yes	yes	yes	no	no	yes
HVTN, IAVI	no	yes	yes	no	no	no	yes
HVTN	no	yes	yes	yes	yes	no	no
INDEPTH	yes	no	yes	yes	no	no	yes
INDEPTH	yes	yes	yes	yes	yes	no	yes
	no	no	yes	yes	no	yes	yes
HVTN	no	yes	yes	no	no	no	yes
IAVI, INDEPTH	yes	yes	yes	yes	no	yes	yes

Supplementary Table 3

HVTN	no	yes	yes	no	no	yes	yes
INDEPTH	yes	no	no	no	no	no	no
HVTN	no	yes	yes	yes	no	yes	yes
IAVI	no	no	no	no	no	no	yes
HVTN	no	yes	yes	yes	no	no	yes

Supplementary Table 3

Parasitology	Immunology	Epidemiology	Genetics	Modeling	Health economics	Health policy/health systems	Social science
Yes	No	Yes	No	No	no	no	no
Yes	No	Yes	No	No	yes	yes	yes
yes	No	Yes	No	No	no	yes	yes
Yes	No	Yes	Yes	No	yes	yes	yes
Yes	No	Yes	No	No	no	no	no
Yes	No	Yes	No	No	no	no	no
yes	No	Yes	No	No	yes	yes	yes
No	No	Yes	No	No	no	no	no
Yes	No	Yes	No	No	no	no	no
Yes	Yes	Yes	No	No	yes	yes	yes
Yes	yes	Yes	no	no	no	no	no
Yes	no	Yes	yes	no	yes	yes	yes
Yes	no	Yes	no	no	yes	yes	yes
No	No	No	no	no	no	no	no
No	No	Yes	no	no	no	no	no
Yes	No	No	no	no	no	no	no
Yes	Yes	Yes	yes	yes	yes	yes	yes
yes	yes	yes	yes	yes	yes	yes	yes
yes	no	yes	no	no	no	no	no
no	no	yes	no	no	yes	yes	yes

Supplementary Table 3

yes	yes	yes	no	no	no	yes	yes
yes	no	yes	no	no	no	no	no
yes	yes	yes	no	no	no	no	no
yes	yes	yes	no	yes	no	no	yes
no	no	yes	no	no	no	no	yes
yes	no	yes	no	no	yes	yes	yes
yes	no	yes	no	no	no	no	no
no	no	yes	no	yes	no	no	no
no	no	yes	no	no	no	no	no
yes	no	yes	no	yes	yes	yes	yes
no	no	yes	no	no	no	no	no
yes	no	yes	no	no	no	no	no
no	no	yes	no	no	no	no	no
no	yes	yes	no	no	no	no	yes
no	yes	yes	no	no	no	no	no
yes	no	yes	no	no	no	no	no
no	yes	yes	no	yes	no	yes	yes
no	yes	yes	no	no	no	yes	yes
no	yes	yes	no	no	no	no	yes
no	yes	yes	no	no	yes	yes	yes
no	no	yes	no	no	no	no	yes
no	yes	yes	no	yes	yes	yes	yes
yes	no	yes	no	yes	no	yes	yes
yes	yes	yes	no	no	no	no	yes
no	yes	yes	no	no	no	no	no
no	yes	yes	no	no	no	yes	yes

Supplementary Table 3

no	yes	yes	no	no	no	no	no
no	no	yes	no	no	no	no	yes
no	no	yes	no	no	yes	yes	yes
no	yes	yes	no	no	no	no	yes
no	yes	yes	no	no	no	no	yes
