# Supplementary material for: Vaccinology in sub-Saharan Africa
Source: BMJ Glob Health. 2019 Sep 20;4(5):e001363. doi: 10.1136/bmjgh-2018-001363 (PMC6768329; doi:10.1136/bmjgh-2018-001363)
Supplement: Supplementary data [file bmjgh-2018-001363supp011.pdf]

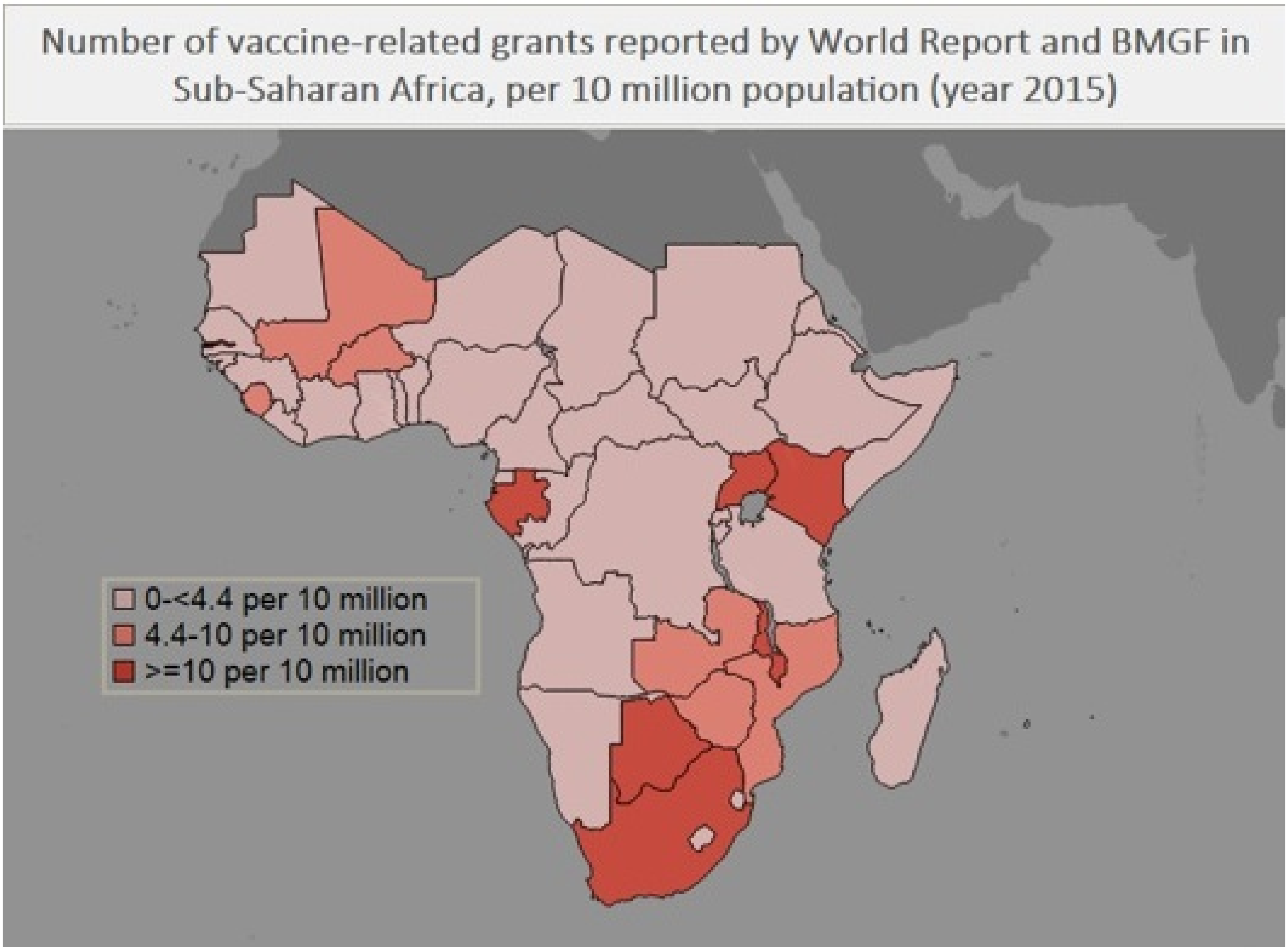
Number of vaccine-related grants reported by World Report and BMGF in Sub-Saharan Africa, per 10 million population (year 2015)
0-<4.4 per 10 million
4.4-10 per 10 million
>=10 per 10 million
