# Supplementary material for: Vaccinology in sub-Saharan Africa
Source: BMJ Glob Health. 2019 Sep 20;4(5):e001363. doi: 10.1136/bmjgh-2018-001363 (PMC6768329; doi:10.1136/bmjgh-2018-001363)
Supplement: Supplementary data [file bmjgh-2018-001363supp004.pdf]

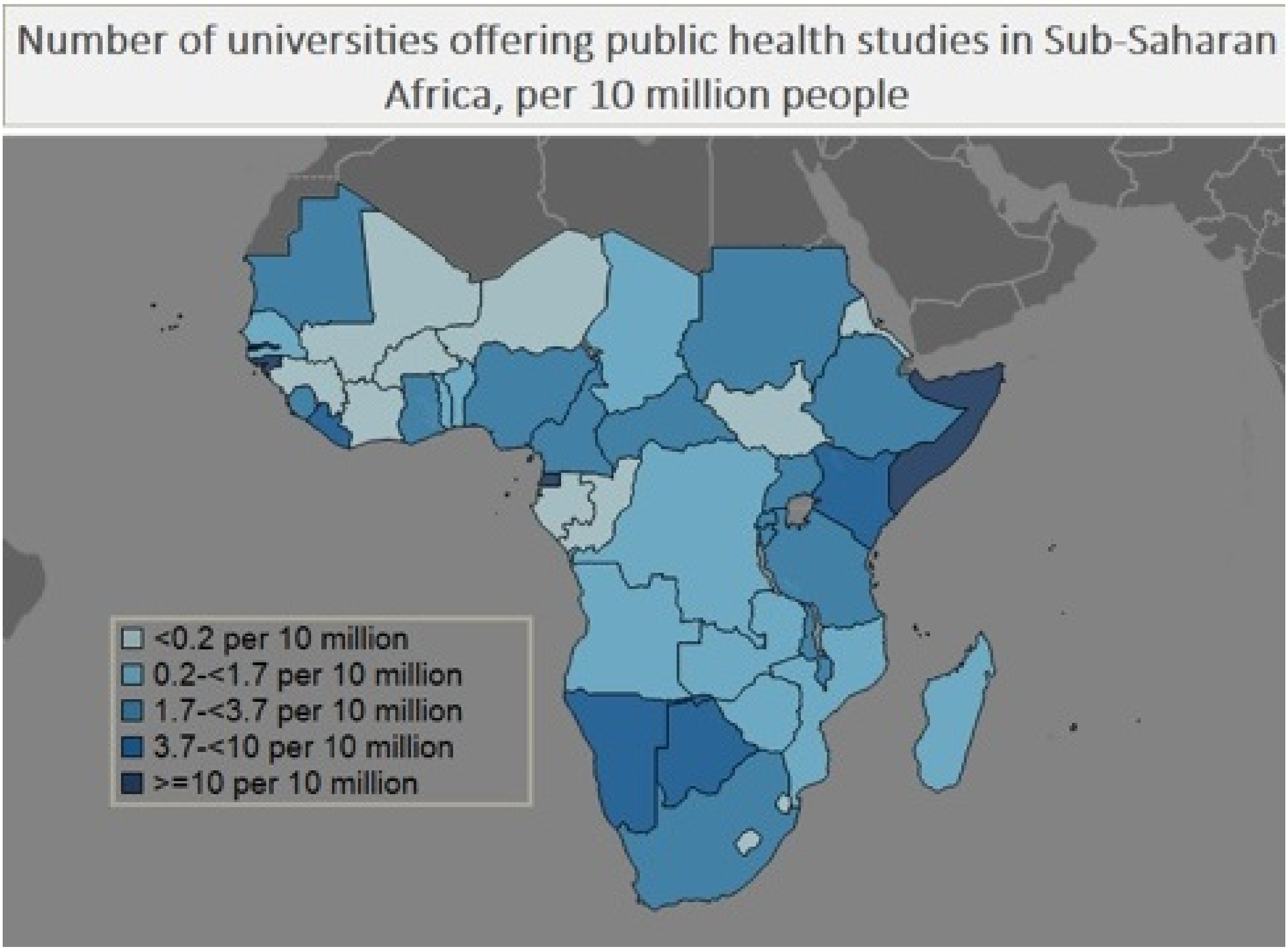
Number of universities offering public health studies in Sub-Saharan Africa, per 10 million people
<0.2 per 10 million
0.2-<1.7 per 10 million
1.7-<3.7 per 10 million
3.7-<10 per 10 million
>=10 per 10 million
